# FOXK2 targeting by the SCF-E3 ligase subunit FBXO24 for ubiquitin mediated degradation modulates mitochondrial respiration

**DOI:** 10.1016/j.jbc.2024.107359

**Published:** 2024-05-10

**Authors:** Rabab El-Mergawy, Lexie Chafin, Jose A. Ovando-Ricardez, Lorena Rosas, MuChun Tsai, Mauricio Rojas, Ana L. Mora, Rama K. Mallampalli

**Affiliations:** Division of Pulmonary, Critical Care, and Sleep Medicine, Department of Internal Medicine, The Ohio State University, Columbus, Ohio, USA

**Keywords:** FOXK2, ubiquitylation, FBXO24, mitochondria, degradation

## Abstract

FOXK2 is a crucial transcription factor implicated in a wide array of biological activities and yet understanding of its molecular regulation at the level of protein turnover is limited. Here, we identify that FOXK2 undergoes degradation in lung epithelia in the presence of the virulent pathogens *Pseudomonas aeruginosa* and *Klebsiella pneumoniae* through ubiquitin-proteasomal processing. FOXK2 through its carboxyl terminus (aa 428–478) binds the Skp-Cullin-F-box ubiquitin E3 ligase subunit FBXO24 that mediates multisite polyubiquitylation of the transcription factor resulting in its nuclear degradation. FOXK2 was detected within the mitochondria and targeted depletion of the transcription factor or cellular expression of FOXK2 mutants devoid of key carboxy terminal domains significantly impaired mitochondrial function. In experimental bacterial pneumonia, *Fbxo24* heterozygous mice exhibited preserved mitochondrial function and Foxk2 protein levels compared to WT littermates. The results suggest a new mode of regulatory control of mitochondrial energetics through modulation of FOXK2 cellular abundance.

Pathogens utilize a multitude of mechanism to undermine host metabolic, replicative, protein synthetic, and innate immune functions that impair cellular viability (1, 2, 3, 4, 5). Among these potential alterations, their impact on host transcription factors is multifaceted and can involve both activation and suppression of levels of various transcriptional programs (5, 6, 7). Changes in the transcriptional machinery play a crucial role in both host defense mechanisms and in the pathogen’s ability to establish and maintain infection (5, 8). For example, nuclear factor erythroid 2 related factor 2 (Nrf2) and activator protein-1 are regulated by viral infections (9, 10, 11, 12). The bacterial pathogen, *Pseudomonas aeruginosa* manipulates host transcription factors to facilitate infection and evasion of host immune responses to modulate its virulence (8, 13, 14). These effects of *P. aeruginosa,* in part, involve regulating activities of hypoxia-inducible factor-1α (15, 16) or peroxisome proliferator-activated receptor gamma (PPAR-γ) (16, 17, 18, 19) to promote a more favorable environment for bacterial survival and replication within host cells (14). The effect of microbial actions on many transcriptional networks requires further investigation.

The forkhead box (FOX) proteins constitute a family of transcription factors characterized by their well-preserved fork-head and winged-helix DNA binding domains, which display a specific affinity for the universally conserved DNA sequence 5ʹ-TTGTTTAC-3ʹ (20, 21, 22). FOXK, comprising FOXK1 and FOXK2, harbor a forkhead-associated (FHA) domain rich in phospho-threonine-residues that distinguishes it from other FOX transcription factors (23). FOXK2 also contains a nuclear localization signal that engages DNA *via* a forkhead domain (FHD). FOXK1 and FOXK2 display ubiquitous expression at both the RNA and protein levels to regulate cell proliferation, survival, skeletal muscle regeneration, differentiation, and tumor progression (24, 25, 26, 27, 28, 29, 30). Despite a high degree of structural and functional conservation, the regulatory functions of FOXK1 and FOXK2 are nonredundant biologically (25, 26, 29, 30, 31). For example, FOXK1 has been implicated in promoting cell proliferation and tumorigenesis in breast cancer and hepatocellular carcinoma (32, 33). In contrast, FOXK2 exerts tumor-suppressive functions in prostate cancer and medulloblastoma (23). Additionally, FOXK1 appears to regulate expression of genes involved in glucose metabolism, whereas FOXK2 regulates the DNA damage response and its repair (34, 35).

In the lung, FOX proteins serve crucial functions in coordinating lung development and homeostasis where their depletion during microbial infections result in uncontrolled goblet cell hyperplasia and metaplasia, altered mucus secretion, and impaired mucociliary clearance of pathogens (36, 37, 38). However, the current understanding of the impact of microbes on members of the FOX family remains limited.

FOXK2 exerts a dynamic role in transcriptional regulation that is context-dependent, where it can act as a transcriptional activator or repressor, shaping the expression profiles of genes involved in diverse cellular functions (35, 39, 40). FOXK2 exhibits versatility in interacting with an array of transcription factors, cofactors, and regulatory proteins to effectively regulate gene expression. Similarly, FOXK2 activates hypoxia-inducible factor-1α to control glucose, serine, and nucleotide metabolism and interacts with other transcriptional factors including AP-1 (27, 41), β-catenin (42, 43), or proliferator-activated receptor-gamma coactivator 1α (PGC-1α) (28) to affect a wide array of cellular processes. FOXK2 also influences chromatin histone acetylation and DNA methylation (31, 40, 44) involved in tumorigenesis (27, 29, 35, 39). Collectively, these observations underscore the premise that cellular concentrations of FOXK2 protein levels may be crucial in determining its transcriptional behavior in diverse settings.

Ubiquitylation is a highly dynamic and tightly regulated process that controls protein levels with relative precision (45, 46, 47, 48). Substrate ubiquitylation occurs through a finely orchestrated sequence of events involving an ubiquitin-activating enzyme (E1), an ubiquitin-conjugating enzyme (E2), and finally conjugation of ubiquitin to a target protein catalyzed by an ubiquitin ligase (E3). The S-phase kinase-associated protein 1 (Skp1)-Cullin 1 (CUL)-F-box (SCF) protein complex represents a prototypical multicomponent subfamily of CUL-RING E3 ligases, containing a crucial substrate receptor component, the F-box protein (49, 50, 51). Within the SCF complex, the F-box protein first engages with the substrate *via* its carboxy-terminal substrate binding domain and subsequently binds to Skp1 through its N-terminal F-box domain (52, 53). To date, the molecular control of FOXK proteins by the ubiquitin apparatus is largely unknown.

In this study, we demonstrate that FOXK2 is depleted in lung epithelial cells after bacterial infection and that the transcription factor is targeted for disposal in cells by the SCF^FBXO24^ ubiquitin E3 ligase. FOXK2 ubiquitylation and degradation triggered by FBXO24 occurs *via* specific molecular signatures that impact mitochondrial function. Specifically, carboxy-terminal domains residing within FOXK2 are crucial in preserving cellular energetics. The results suggest that FBXO24 may play an important role in impairing energy stores within cells through reduced FOXK2 abundance and its transcriptional activities.

## Results

### Bacterial targeting of FOXK2 for degradation through ubiquitin-proteasomal processing

**Figure 1:**
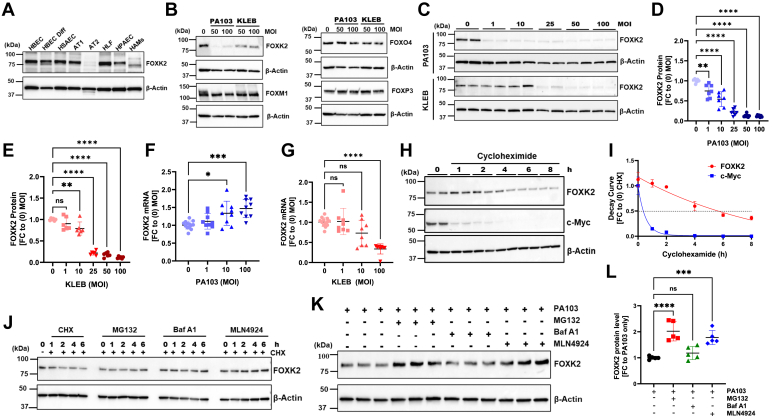
**Bacteria target FOXK2 for ubiquitin-proteasomal degradation.***A*, FOXK2 protein levels in various human lung primary cells. *B*, representative immunoblot showing protein levels of members of the Forkhead box (FOX) family in BEAS-2B cells infected with various multiplicity of infection (MOI) of *Pseudomonas aeruginosa* (PA103) or *Klebsiella pneumoniae* (KLEB) for 6 h. *C*, FOXK2 protein levels in BEAS-2B cells following various MOI of PA103 or KLEB infection for 6 h. *D*-*E*, densitometric quantification of immunoblot data from panel (*C*) for PA103 infection (*D*) and KLEB infection (*E*). Data are expressed as fold-change (FC) relative to MOI = 0. *F*-*G*, FOXK2 mRNA levels in BEAS-2B cells after PA103 infection (*F*) or KLEB infection (*G*) for 6 h assayed using qPCR. *H*, FOXK2 and c-Myc half-life in BEAS-2B cells using cycloheximide (50 μg/ml) treatment for up to 8 h. *I*, FOXK2 decay curve in comparison to c-Myc from the immunoblot in panel (*H*). *J*, FOXK2 protein levels in BEAS-2B after treatment with cycloheximide (50 μg/ml), with or without MG132, bafilomycin A1 (Baf A1) or MLN4924 for 6 h, showing no changes over time under native conditions. *K*, BEAS-2B cells were treated with MG132 (50 μM), bafilomycin A1 (20 μM), or MLN4924 (10 μM) in the presence of PA103 (10 MOI) for 6 h, followed by immunoblotting. Data show accumulation of FOXK2 after PA103 infection with incubation of MG132 or MLN4924. *L*, densitometry of immunoblot results from panel (*K*). For panels (*A*–*B*), results are from *n* = 2 separate experiments, panels (*C*–*G*), each dot represents an individual experiment, panels (*H*–*L*), results are from *n* = 2 to 3 separate experiments. ∗∗∗∗*p* < 0.0001, ∗∗∗*p* < 0.001, ∗∗*p* < 0.01 and ∗*p* < 0.05. The densitometric analysis involved calculating the fold change (FC) after normalizing the immunoblot signals to the loading control. Statistical analysis was by one-way ANOVA. FOX, forkhead box; qPCR, quantitative PCR.

We first evaluated FOXK2 protein levels in various primary human lung cells to localize its predominant expression. The data revealed robust expression of FOXK2 in human lung epithelia, particularly in human undifferentiated and differentiated (Diff) bronchial epithelial cells (HBEC) and human small airway epithelial cells (HSAEC), in contrast to immune effector cells such as human alveolar macrophages (Fig. 1*A*). Further, our data indicate significant expression of FOXK2 in AT1 cells but nearly undetectable in AT2 cells. High-level AT1 cell expression may be associated with FOXK2’s putative role in gas-exchange although this requires investigation. To understand the impact of microbial infection on these epithelia, we infected a human bronchial epithelial cell line, BEAS-2B, with either of the two gram-negative pathogens, *P. aeruginosa* or *Klebsiella pneumoniae*. When we assayed protein levels of various FOX family members, only FOXK2 exhibited a significant reduction in protein levels compared to other members (Fig. 1*B*). Effects of these pathogens on FOXK1 were inconsistent (data not shown). Both *P. aeruginosa* and *K. pneumoniae* generally triggered a substantial reduction in steady-state FOXK2 protein levels dependent on the bacterial load (Fig. 1, *C*–*G*). While *P. aeruginosa* (strain PA103) infection of cells reduced FOXK2 protein as the multiplicity of infection (MOI) increased, there was a notable increase in FOXK2 mRNA levels, perhaps representing a compensatory mechanism (Fig. 1, *D* and *F*). In contrast, *K. pneumoniae* induced a modest decrease in both protein and mRNA levels only at higher MOI (Fig. 1, *E* and *G*). Interestingly, gram-positive bacteria such as *Staphylococcus aureus* did not impact FOXK2 protein or mRNA levels (Fig. S1). Thus, some gram-negative bacterial pathogens differentially modulate FOXK2 protein and mRNA levels. The observation that *P*. *aeruginosa* infection reduced protein levels of FOXK2 without reduced mRNA suggests an effect at the posttranslation level, but the data do not exclude effects on mRNA translational efficiency.

To assess protein stability of FOXK2, BEAS-2B cells were treated with the protein biosynthesis inhibitor cycloheximide (CHX) over a time course, followed by immunoblotting. The results revealed that in comparison to c-Myc, FOXK2 exhibited stability with a half-life (t ½) of approximately 4 to 5 h (Fig. 1, *H* and *I*). Next, cells were treated with a proteasome inhibitor (MG132), a lysosome inhibitor (Bafilomycin A1) or a Cullin-RING E3 ligase inhibitor (MLN4924) in the presence of CHX. Surprisingly, under the experimental conditions tested, we were not able to confidently determine the pathway by which FOXK2 is degraded (Fig. 1*J*). However, in the presence of *P*. *aeruginosa* infection there was a significant increase in FOXK2 protein accumulation when cells were treated with the proteasome inhibitor, or by the Cullin-RING E3 ligase inhibitor (Fig. 1, *K* and *L*). These findings suggest that *P. aeruginosa* infection triggers the degradation of FOXK2 *via* the ubiquitin-proteasomal pathway, suggesting a potential role for CUL-RING E3 ligases in modulating availability of the transcription factor during bacterial infection.

### FBXO24-mediated degradation of FOXK2 protein

**Figure 2:**
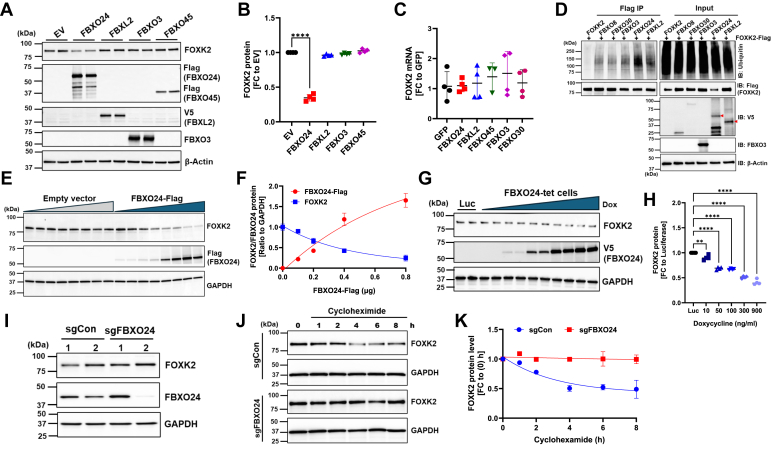
**FBXO24 mediates FOXK2 degradation.***A*, BEAS-2B cells were transfected with various tagged F-box proteins followed by immunoblotting for indicated proteins. EV, empty vector. All F-box proteins for detection are Flag-tagged at the carboxyl terminus, with the exception of FBXO3 (no tag). *B*, densitometry of the immunoblot results in panel (*A*). *C*, *FOXK2* steady-state mRNA levels in BEAS-2B cells-expressing various ectopically expressed F-box proteins showing no change in mRNA levels. *D*, coimmunoprecipitations (Co-IP). Cells were cotransfected with *FOXK2*-Flag and one of the indicated V5-tagged F-box protein encoding-plasmids, followed by Flag (FOXK2) immunoprecipitation and immunoblotting. *E*, BEAS-2B cells were transfected with increasing amounts of an empty vector or *FBXO24*-Flag plasmid (0, 0.2, 0.4, or 0.8 μg, each dose is represented by two replicates) followed by immunoblotting (*E*) and densitometry (*F*). *G*, representative immunoblot and (*H*) densitometry in tetracycline inducible-FBXO24 BEAS-2B cells treated with increasing amounts of doxycycline (Dox) and a luciferase (Luc) control. In (*G*) each pair of lanes from left show levels of FOXK2 protein after one Dox concentration (10, 50, 100, 300, or 900 ng/ml) showing an inverse correlation between FOXK2 and FBXO24 protein levels in comparison to Luc-control expressing cells (Luc cells were treated with 900 ng/ml, represented by two replicates on far-left lanes in [G]). *I*, immunoblot of FOXK2 protein levels in CRISPR/Cas9 knockout FBXO24-BEAS-2B cells (sgFBXO24) and in CRISPR control (sgCon) cells. *J*, immunoblot and corresponding densitometry (*K*) showing FOXK2 protein stability in sgFBXO24-BEAS-2B *versus* sgCon following treatment with cycloheximide (50 μg/ml) for 8 h. For all panels, results represent 3 to 4 separate experiments. ∗∗∗*p* < 0.001 and ∗∗*p* < 0.01. The densitometric analysis involved calculating the fold change (FC) after normalizing the immunoblot signals to the loading control. Statistical analysis was by one-way ANOVA. FOX, forkhead box.

In separate studies, we used an unbiased proximity-dependent biotinylation assay to assess potential substrates for the F-box protein, FBXO24. When comparing Wt FBXO24 with a catalytically inactive FBXO24 mutant (ΔLPAA), FOXK2 was found to be more abundant in cells expressing the ΔLPAA mutant than in those expressing the Wt FBXO24 (54). Hence, we postulated that FBXO24 mediates FOXK2 degradation through SCF^FBXO24^ E3 ligase polyubiquitylation. Indeed, after overexpressing various F-box plasmids within the SCF family in BEAS-2B cells, only *FBXO24*-expressing cells exhibited a marked decrease in FOXK2 protein levels (Fig. 2, *A* and *B*) with no effect on steady-state mRNA levels (Fig. 2*C*). In these studies, we cannot exclude that some variability in F-box plasmid expression harboring the Flag tag might account for the observations on FOXK2 degradation. Next, in coimmunoprecipitation (Co-IP) studies where we coexpressed *FOXK2*-Flag with various plasmids encoding F-box proteins, immunoblotting using ubiquitin antibody in Flag-pull downs revealed the highest level of polyubiquitylation when *FOXK2* was coexpressed with *FBXO24* plasmid (Fig. 2*D*). BEAS-2B cells transfected with increasing concentrations of *FBXO24*-Flag plasmid triggered reduced endogenous FOXK2 protein levels and a robust increase in ectopically expressed FBXO24 protein compared to cells transfected with *GFP* control plasmid without changes in steady-state FOXK2 mRNA (Figs. 2, *E* and *F*, and S2*A*). Similar results were obtained in tetracycline-inducible *FBXO24*-V5 expressing cells (Fig. 2, *G* and *H*), confirming that FOXK2 degradation in FBXO24-expressing cells occurs in a dose-dependent manner, with no discernible effect on mRNA levels (Fig. S2*B*). Moreover, transfection of BEAS-2B cells with increasing amounts of a plasmid encoding a catalytically inactive FBXO24 mutant (ΔLPAA) did not result in any change in FOXK2 protein levels compared to cells transfected with a control plasmid (Fig. S2*C*). We used CRISPR/Cas9 technology to stably deplete *FBXO24* from BEAS-2B cells and expanded a cell line sufficiently devoid of FBXO24 with modestly increased FOXK2 levels (Fig. 2, *I* and *J*). Using these sg*FBXO24* stably deficient cells in CHX chase experiments we observed that depleting FBXO24 protein significantly extended the lifespan of FOXK2 compared to control cells (Fig. 2, *J* and *K*). Collectively, these findings strongly suggest that FBXO24 plays a key role in affecting FOXK2 protein concentrations by destabilizing levels of the transcription factor.

### Multiple acceptor sites exist for FOXK2 polyubiquitylation

**Figure 3:**
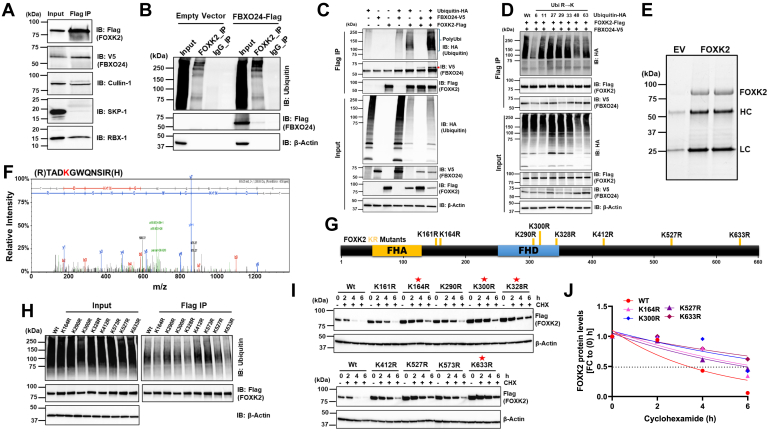
**FOXK2 is polyubiquitylated by SCF**^**FBXO24**^**E3 ligase complex.***A*, BEAS-2B cells were cotransfected with *FOXK2*-Flag and *FBXO24*-V5 plasmids, followed by Co-IP using Flag beads to pull down FOXK2-Flag and immunoprecipitants probed for SCF components (*n* = 3). *B*, Beas2B cells were transfected with an empty vector or a *FBXO24*-Flag plasmid, endogenous FOXK2 was immunoprecipitated with a primary antibody, and immunoprecipitants probed with ubiquitin antibody showing increased polyubiquitylation of endogenous FOXK2. IgG was used as a control. (*n* = 3) (*C*) BEAS-2B cells were transfected with the indicated plasmids followed by Flag (FOXK2-Flag) bead immunoprecipitation and immunoblotting showing increased polyubiquitylation of ectopically expressed FOXK2-Flag (*n* = 3). *D*, BEAS-2B cells were cotransfected with *FOXK2*-Flag and *FBXO24*-V5 plasmids, with one of various plasmids encoding *ubiquitin*-HA R→K mutants, followed by Flag immunoprecipitation and immunoblotting (*n* = 3). *E*, stain-free SDS-PAGE gel showing immunoprecipitated FOXK2-Flag prior to processing for on-beads digestion to identify ubiquitylated lysine(s) using MS analysis (*n* = 4). *F*, a representative MS peptide spectrum showing mapping sites of lysine ubiquitylation within FOXK2-peptides in BEAS-2B cells. The displayed fragment harboring a lysine residue, K290, was randomly chosen for display above. *G*, FOXK2 schematic diagram map showing FHA (forkhead associated), FHD (forkhead DNA binding) domains and positions of MS-identified ubiquitylated lysine residues. *H*, cells were transfected with K→R mutant *FOXK2*-Flag plasmids, proteins pulled down using Flag beads, and processed for ubiquitin immunoblotting (*n* = 3). *I*, cells were transfected with K→R mutant *FOXK2*-Flag plasmids as above and incubated with cycloheximide (CHX) (50 μg/ml) for up to 6 h to determine protein half-life in BEAS-2B cells (*n* = 4). *Red stars* indicate stabilized mutants. *J*, decay curves for selected lysine mutants compared to FOXK2 Wt from panel (*I*). The densitometric analysis involved calculating the fold change (FC) after normalizing the immunoblot signals to the loading control. FHD, forkhead domain; FOX, forkhead box; IgG, immunoglobulin G; SCF, S-phase kinase-associated protein 1 (Skp1)-Cullin 1 (CUL)-F-box.

To investigate interaction between FOXK2 and the SCF ubiquitin machinery, we conducted Co-IPs using FOXK2-Flag, followed by immunoblotting of key E3 ubiquitin ligase components in the immunoprecipitants. FOXK2 interacts with several critical SCF components, including FBXO24, Cullin1, and RBX1 (Figs. 3*A*, and S3). In addition, we evaluated the impact of *FBXO24* overexpression on FOXK2 ubiquitylation levels in BEAS-2B and in HEK-293T cells followed by immunoprecipitation (IP) using either FOXK2 antibody or Flag beads. Immunoblots demonstrated that *FBXO24* cellular overexpression facilitated ubiquitylation of both endogenous substrate (Fig. 3*B*) and upon ectopically expressed *Flag-FOXK2* (Fig. 3*C*). Typically, proteasomal degradation occurs through a K48 linkage, but other types of ubiquitin linkages have been described for cyclin B1 (55) and APC/C (56) through K11. Additionally, LRRK2 is targeted for proteasomal degradation through combination of K27 and K29-linked polyubiquitination (57). Here, FOXK2 polyubiquitylation occurs predominantly through K11, K27, K29, and K33 linkages (Fig. 3*D*). This intriguingly suggests that FBXO24-mediated polyubiquitylation of FOXK2 may extend beyond its role in stability regulation potentially serving as a versatile signaling mechanism that impacts FOXK2 functions. In separate experiments, we investigated potential FOXK2 acceptor site(s) for ubiquitylation. To achieve this, *FOXK2*-Flag was coexpressed with *FBXO24*-V5 in BEAS-2B cells, followed by Flag IP and posttranslational modification analysis using liquid chromatography with tandem mass spectrometry (LC-MS/MS) (Fig. 3, *E* and *F*). Mass spectrometry (MS) analysis identified 11 lysine (K) residues within FOXK2 as potential ubiquitylation targets (Fig. 3*F*). Subsequently, each of these K residues shown in Figure 3*G* were substituted with arginine (R) and *FOXK2* variant plasmids ectopically expressed in BEAS-2B or HEK-293T cells to assess their stability and ubiquitylation levels. Flag (FOXK2) IP studies revealed that four lysine mutants, K^164R^, K^300R^, K^328R^, and K^633R^, exhibited reduced polyubiquitylation levels compared to the Wt FOXK2 protein, implying that one or more of these residues likely serves as FBXO24-dependent ubiquitin acceptor sites within FOXK2 (Fig. 3*H*). This observation was further substantiated by assessing the degradation of these mutants in the presence of CHX. Notably, when expressed in cells the K^164R^, K^300R^, K^328R^, and K^633R^ mutants displayed significantly enhanced protein stability compared to the Wt and other K mutants (Figs. 3, *I* and *J*, and S4). The data do not exclude other potential ubiquitin acceptor sites (*e.g.*, K^573^). Thus, multiple lysine linkages and acceptor sites were uncovered within FOXK2 in response to FBXO24 mediated ubiquitin chain polyubiquitylation.

### FOXK2 displays critical biological responses in lung epithelia

**Figure 4:**
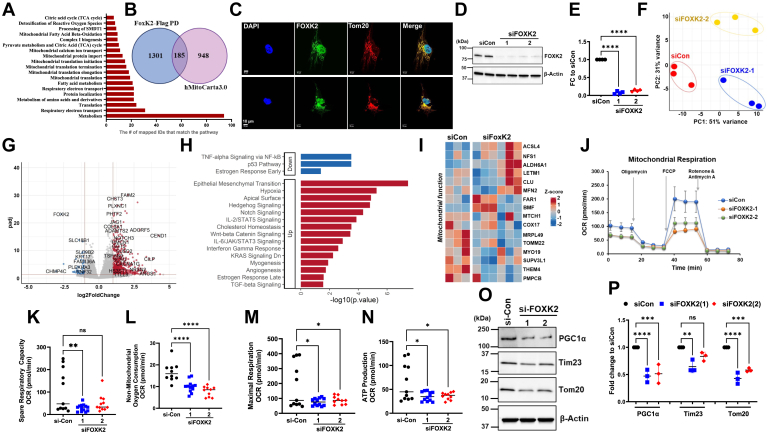
**FOXK2 exhibits diverse biological roles in lung epithelia.***A* and *B*, proteome analysis with a Venn diagram and Reactome pathways analysis of mitochondrial proteins identified in FOXK2 pull downs. *C*, BEAS-2B cells were subjected to immunofluorescence staining with anti-FOXK2, anti-Tom20, and DAPI, indicating colocalization of endogenous FOXK2 with DAPI in the nucleus and with Tom20 in the mitochondria. Two different frames are on display as shown by each row. The scale bar represents 10 μm. *D*, immunoblot and its densitometry (*E*) confirming successful knockdown of *FOXK2* in BEAS-2B cells transfected with predesigned *FOXK2*-specific siRNAs compared to cells transfected with scrambled siRNA. These cells were used for Seahorse assays below. *F*, principal component analysis plots of the transcriptome profiles of cells transfected with siRNA control (siCon) (*red*), or either one of two different siRNA against *FOXK2*; *siFOXK2-1* (*blue*), or si*FOXK2*-*2* (*yellow*). *G*, volcano plots showing the distribution of differentially regulated genes with log-fold change as expressed *versus* control after *FOXK2* knockdown in cells. *H*, pathway analysis of top upregulated and downregulated genes in *FOXK2* knockdown cells using the M SigDB Hallmark database through the online platform enrichR. *I*, heatmap of mitochondrial-related genes in control (siCon) *versus FOXK2* knockdown cells. *J*–*N*, mitochondrial function as assessed by measurement of the oxygen consumption rate (OCR) in siCon *versus FOXK2*-knockdown cells using a Seahorse XFe96 bioanalyzer. Readouts include mitochondrial oxidative phosphorylation activity (*J*), spare respiratory capacity (*K*), nonmitochondrial oxygen consumption (*L*), maximal respiration (*M*), and ATP production (*N*). *O*, BEAS-2B lysates from panel (*D*) were used to assess protein levels of mitochondrial markers, indicating reduced protein levels of PGC1α, Tom20, and Tim23 in *FOXK2*-knockdown cells *versus* control cells and results quantitated in (*P*) (*n* = 3). Data are mean ± SEM (One-way ANOVA) ∗∗∗*p* < 0.001 ∗∗*p* < 0.01 and ∗*p* < 0.05. DAPI, 4′,6-diamidino-2-phenylindole; FOX, forkhead box.

To delve deeper into FOXK2 physiological roles, we ectopically expressed *FOXK2*-Flag in BEAS-2B cells and utilized MS to identify FOXK2-associated proteins (Fig. 4*A*). The analysis unveiled various proteins within the FOXK2 pull-downs linked to a wide range of pathway activation as determined by gene ontology criteria (Table S1). To validate this, all identified proteins from FOXK2 pull-down were cross-referenced with the human MitoCarta database (hMitoCarta3.0) (58), which pinpointed strikingly 185 proteins with mitochondria-related functions (Fig. 4*B*). Further, Reactome pathway analysis was conducted to categorize these proteins based on their biological roles (Fig. 4*A*). Intriguingly, the largest subset of identified proteins was found to be associated with metabolism, followed by proteins involved in respiratory electron transport and translation. The substantial number of mitochondrial proteins interacting with FOXK2, whether directly or indirectly, also raised the possibility of FOXK2 localization within the mitochondria. To investigate this, BEAS-2B cells were stained with FOXK2 antibody to determine subcellular localization of endogenous FOXK2. Cells underwent immunofluorescence staining using anti-FOXK2 and anti-Tom20 as a mitochondrial marker. The observed colocalization of FOXK2 and Tom20 provided evidence that FOXK2 indeed localizes within the mitochondria, in addition to its presence in the nucleus (Fig. 4*C*).

To further define the biological role of FOXK2, *FOXK2* specific-siRNA was used to deplete *FOXK2* in BEAS-2B cells. The knockdown was confirmed by immunoblot analysis (Fig. 4, *D* and *E*) and qPCR (Fig. S5). Total mRNA from these cells was subjected to RNAseq analysis using an Illumina NovaSeq platform. Principle component analysis demonstrated global changes in gene expression after *FOXK2* cellular silencing. In the basal state of over 15,077 genes were identified after FOXK2 silencing (Fig. 4, *F* and *G*). Gene set analysis revealed changes in several cellular pathways after *FOXK2* knockdown in epithelia. Notably, genes associated with p53 and tumor necrosis factorα (*via* NF-κB) pathways were downregulated in *FOXK2*-knockown cells (Fig. 4*H*). Intriguingly, while *FOXK2* knockdown resulted in the downregulation of the early estrogen response pathway, it conversely upregulated the late estrogen response pathway (Fig. 4*H*). Genes that showed the most significant upregulation in *FOXK2* knockdown cells were primarily linked to the epithelial-mesenchymal transition pathway, followed by genes associated with hypoxia and hedgehog signaling (Figs. 4, *G* and *H*, and S6). Additionally, *FOXK2* knockdown led to the upregulation of several oncogenic pathways, including Notch, Wnt/β-catenin, Kirsten rat sarcoma virus (KRAS) and transforming growth factor beta (TGFβ) signaling (Fig. 4*H*). Collectively, these results indicate that FOXK2 may have diverse roles in suppressing oncogenic pathways, inflammatory responses to microbial pathogens, and cellular homeostasis in lung epithelia.

### FOXK2 regulates mitochondrial function

Consistent with FOXK2 pull-down data analysis, transcriptomic profiling of *FOXK2*-knockdown cells demonstrated substantial alterations in genes related to protein translation, biogenesis, and the respiratory electron transport (Figs. 4, *F*–*I*, and S6). To further validate FOXK2 as a regulator of mitochondrial function, studies were conducted in *FOXK2*-knockdown BEAS-2B cells using Seahorse assays to measure the oxygen consumption rate (OCR) using two distinct siRNAs targeting *FOXK2* (Fig. 4, *J*–*N*). Notably, using these two siRNAs, the *FOXK2* knockdown cells exhibited pronounced reductions in OCR and spare respiratory capacity (Fig. 4, *J* and *K*). Additionally, we observed moderate reductions in nonmitochondrial oxygen consumption, maximal respiration, and ATP production (Fig. 4, *L*–*N*). Thus, FOXK2 appears indispensable in maintaining cellular metabolism and energy production in comparison to cells transfected with scrambled RNA.

To validate the efficacy of the knockdown, we performed immunoblotting on cell lysates and stained for FOXK2 (Fig. 4, *D* and *O*), as well as other relevant markers including PGC1α, Tim23, and Tom20. The immunoblot confirmed that siRNA-mediated knockdown resulted in depletion of more than 90% of FOXK2 protein levels (Fig. 4, *D* and *E*) and approximately 80 to 90% of mRNA levels (Fig. S5). Importantly, FOXK2-deficient cells exhibited decreased signals in the protein levels of mitochondrial markers Tom20, Tim23 and its biogenesis regulator, PGC1α, when compared to control cells (Fig. 4, *O* and *P*). Collectively, siRNA-mediated knockdown of *FOXK2* in BEAS-2B cells led to substantial alterations in mitochondrial function, accompanied by changes in key mitochondrial markers.

### Mapping the molecular signature of FOXK2 interaction with FBXO24

**Figure 5:**
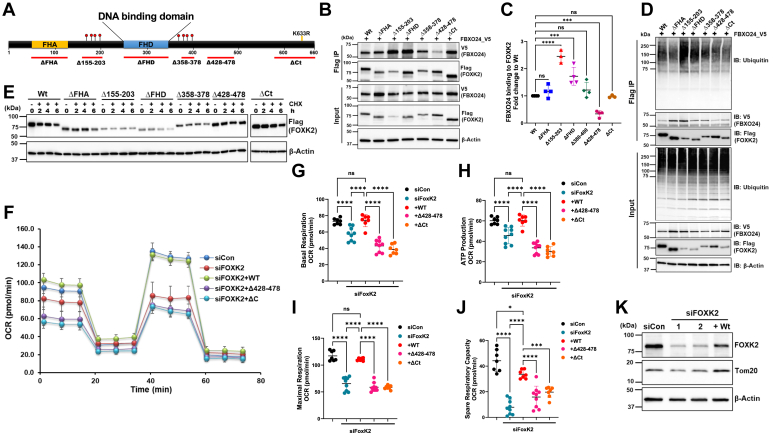
**Molecular signatures within FOXK2 impact FBXO24 binding and mitochondrial respiration.***A*, schematic diagram of FOXK2 internal and carboxy-terminal truncated deletions indicated by *red lines*. *B*, BEAS-2B cells were cotransfected with *FBXO24*-V5 with plasmids encoding *FOXK2*-Flag internal or carboxyl terminal truncated mutants followed by Co-IPs using Flag magnetic beads. Shown are levels of FBXO24 bound to mutant FOXK2 variants after Flag pull-downs (*top blot*) and input (*below*). *C*, densitometric analysis showing fold change of FBXO24 interaction with FOXK2 mutants *versus* Wt from immunoblot results in panel (*B*). *D*, cells were transfected with internal or carboxy-terminal *FOXK2*-Flag plasmids, proteins pulled down using Flag beads, and processed for ubiquitin immunoblotting showing increased polyubiquitylation in Δ155 to 203 and ΔFHD mutants in comparison to FOXK2 Wt. *E*, FOXK2 internal or carboxy-terminal mutant protein half-life was assayed in BEAS-2B cells in presence of CHX for 6 h (*n* = 3). *F*–*J*, BEAS-2B cells depleted of endogenous FOXK2 (siFOXK2) were rescued by transfection with internal or carboxy-terminal truncated mutants and mitochondrial oxidative phosphorylation activity (*F*), basal respiration (*G*), ATP production (*H*), maximal respiration (*I*), and spare respiratory capacity (*J*) are shown. *K*, representative immunoblot confirming FOXK2 knockdown and rescue of both FOXK2 Wt and Tom 20 protein levels in *FOXK2* knockdown BEAS-2B cells (*n* = 3). Data are mean ± SEM (One-way ANOVA), ∗∗∗∗*p* < 0.001 ∗∗*p* < 0.01 and ∗*p* < 0.05. CHX, cycloheximide; FHD, forkhead domain; FOX, forkhead box.

FOXK2 undergoes a potential high-level phosphorylation in various regions: within the FHA domain, between the FHA and FHD domains at aa 160 to 225, and last after the FHD domain at aa 380 to 400 (26, 35) (Fig. 5*A*). Since F-box proteins often target their substrates for degradation through phosphodegrons, we hypothesized that one of these regions serves as a molecular signature critical for the interaction between FOXK2 and FBXO24. To test this, we generated various internal and carboxy terminal truncated deletions in FOXK2, all harboring a Flag tag. Plasmids were coexpressed alongside *FBXO24*-V5, followed by Flag Co-IPs (Fig. 5*B*). Both FOXK2 Wt and deletion variants maintained the ability to bind FBXO24, although to varying degrees (Fig. 5*B*). Unexpectedly, a variant with deletion of the region between aa 428 to 478 (Δ428–478) showed significantly decreased FBXO24 binding compared to FOXK2 Wt (Fig. 5, *B* and *C*), indicating that this region may be necessary for FBXO24 interaction. Conversely, a variant devoid of aa 155 to 203 (Δ155–203) or the FHD domain exhibited increased FBXO24 binding (Fig. 5, *B* and *C*) suggesting that phosphorylation of this domain might protect against FBXO24 interaction with FOXK2, thus impacting vulnerability to its degradation. Further, we executed experiments in which we overexpressed Flag-*FOXK2* with or without *FBXO24*-V5 followed by bacterial infection. The cells were then processed for Flag IP followed by immunoblotting to assess the phosphorylation levels of FOXK2. The data indicate that *FBXO24* overexpression and PA103 infection differentially modulate FOXK2 phosphorylation, depending on the targeted residues. Importantly, the data show that phosphorylation of threonine residues on FOXK2 decreases in the presence of *P. aeruginosa* and FBXO24 (Fig. S7). Thus, although speculative, the aa 155 to 203 (Δ155–203) or the FHD motifs might be prone to site-specific dephosphorylation after PA103 infection thereby altering FOXK2 vulnerability to SCF^FBXO24^ driven degradation.

The observed increased affinity between FBXO24 and one deletion mutant (Δ155–203) or the FHD deletion mutant protein indicates that neither of these regions serves as potential docking sites for FBXO24. In contrast the data suggest that the docking site for FBXO24 likely resides within a stretch of aa 428 to 478 region, as this variant (Δ428–478) exhibited reduced F-box binding and decreased polyubiquitylation whereas the deletion of aa 155 to 203 or the FHD domain demonstrated increased FBXO24 binding and polyubiquitylation (Fig. 5*D*). To validate these observations, we assessed protein stability by subjecting BEAS-2B cells expressing these deletions mutants in cells exposed to CHX treatment (Fig. 5*E*). The results confirmed that the Δ155 to 203 and the FHD variant when expressed in cells degraded faster than the Wt or the other mutants. Conversely, the Δ428 to 478 variant exhibited enhanced stability, supporting the hypothesis that the docking site for FBXO24 is located within the carboxy-terminal region.

### FOXK2 domains impact mitochondrial function

To address the role of the FOXK2 regulatory domains on cellular energetics, we assessed mitochondrial function in FOXK2 depleted BEAS-2B cells using siRNA, and then ectopically expressed *FOXK2* Wt or mutant plasmids into these cells (Figs. 5, *F*–*K*, and S8). Here, si*FOXK2* in cells as before impaired several parameters of mitochondrial function compared to Wt FOXK2. Additionally, deleting the FHA domain showed a moderate reduction in ATP production and basal respiration. Further, expression of plasmids encoding internal deletions of FOXK2 (Δ155–203 and FHD variant) showed significantly reduced maximal respiration and respiratory spare capacity and modestly reduced ATP production than the Wt FOXK2 (Fig. 5, *G*–*J*). This suggests that FOXK2 may regulate some parameters of mitochondrial function indirectly *via* DNA-binding to transactivate mitochondrial-related genes. Intriguingly, the carboxy-terminal truncated mutants including the Δ358 to 378, Δ428 to 478, and Δ588 to 660 (ΔCt) had the most significant impact on disrupting mitochondrial function. In particular, expression of a ΔCt mutant in cells resulted in a substantial reduction in basal respiration, ATP production, maximal respiration, and spare respiratory capacity than cells expressing FOXK2 full length (Fig. 5, *F*–*J*). These findings strongly suggest that the FOXK2 carboxyl terminus might play a crucial role in regulating cellular energetics.

### FOXK2 is degraded within the nucleus

**Figure 6:**
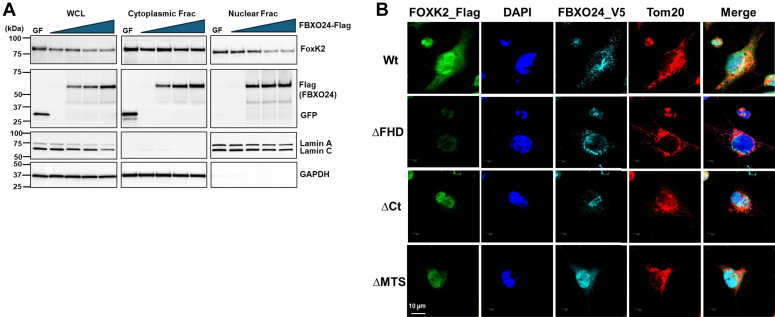
**FBXO24 mediates FOXK2 nuclear degradation.***A*, a representative immunoblot of cytoplasmic and nuclear fractions from BEAS-2B cells transfected with increasing amounts of *FBXO24*-Flag (0.1, 0.2, 0.4, and 0.8 μg, each lane represents one concentration) or *GFP*-V5 (0.8 μg), demonstrating reduced levels of endogenous FOXK2 protein in the nuclear fraction compared to the cytoplasmic fraction. *B*, immunofluorescence staining of FOXK2 in BEAS-2B cells expressing either the Wt or one of the indicated deletion mutants. DAPI, a nuclear marker. BEAS-2B cells were cotransfected with *FOXK2*-Flag Wt or the mutant plasmids, and *FBXO24*-V5, followed by staining with anti-Flag, anti-V5, anti-Tom20, and DAPI, indicating colocalization of FOXK2 Wt and FBXO24 with DAPI in the nucleus and with Tom20 in the mitochondria. Scale bars represent 10 μm, (*n* = 3). DAPI, 4′,6-diamidino-2-phenylindole; FOX, forkhead box.

Prior studies demonstrate various ubiquitin-proteasome and E3 ligase components within the nuclear compartment (59, 60, 61). To address subcellular degradation of FOXK2, ectopically expression of increasing amounts of *FBXO24* plasmid in cells led to a substantial decrease in the mass of the transcription factor in whole cell lysates and within the nuclear fraction (Fig. 6*A*). Limited effects of *FBXO24* plasmid expression were observed on FOXK2 protein in the cytoplasmic fraction. Additional studies were performed to assess the impact of FOXK2 domains on subcellular localization. Here, while Wt FOXK2 when expressed in cells was diffusely present within the cell, ectopic expression of a plasmid devoid of the FHD domain lacked ability for nuclear expression (Fig. 6*B*). In contrast, ectopically expressed plasmids lacking the carboxyl terminus (ΔCt FOXK2 mutant) or a variant lacking a putative mitochondrial targeting signal (ΔMTS, lacking aa 2–29) both were observed to display nuclear signals. Interestingly, although the ΔCt variant is prone to FBXO24-mediated nuclear degradation, when expressed it was stable (Fig. 5*E*) that may be related to its lack of an ubiquitin acceptor site at K^633^. Nevertheless, the data underscore a requirement for a FOXK2 nuclear localization signal (juxtaposed within the FHD) for targeted nuclear degradation catalyzed SCF^FBXO24^.

### FBXO24 regulates mitochondrial function through FOXK2 during *K. pneumoniae* infection

**Figure 7:**
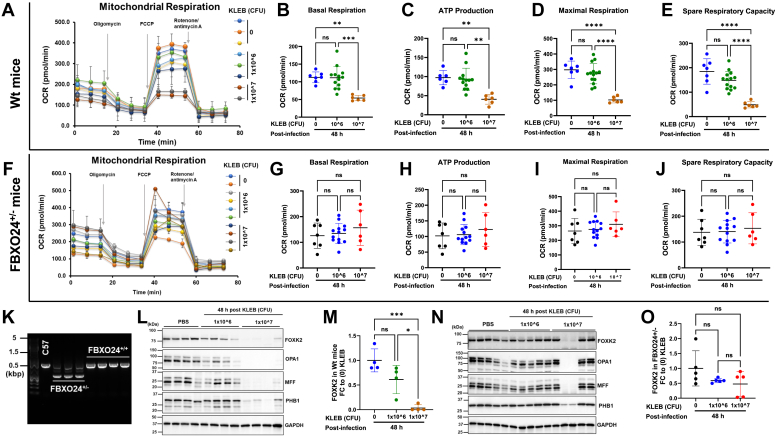
**Preservation of mitochondrial function in Fbxo24 heterozygous mice during experimental bacterial pneumonia.** Wt (*n* = 4 per group) or Fbxo24^+/−^ (*n* = 5 per group) mice were infected with *Klebsiella pneumoniae* (KLEB)/mouse at the indicated cfu/mouse intranasally (i.n.). After 48 h post infection, lung tissue was collected and processed to generate precision-cut lung slices (PCLS). Mitochondrial function was assessed by measuring the oxygen consumption rate (OCR) in PCLS obtained from both PBS and KLEB-infected Wt mice (*A*–*E*) and PBS and KLEB-infected Fbxo24^+/−^ mice (*F*–*J*) using a Seahorse XFe96 bioanalyzer. The OCR readouts included basal respiration (*B* and *G*), ATP production (*C* and *H*), maximal respiration (*D* and *I*), and spare respiratory capacity (*E* and *J*). *K*, shows the confirmation of Fbxo24^+/−^ heterozygous mice *via* genomic PCR analysis in founder mice. *L*, immunoblot of Foxk2, and its densitometry is shown in (*M*), along with various mitochondrial markers protein levels in KLEB-infected Wt mice compared to uninfected mice. *N*, presents an immunoblot of Foxk2, and its densitometry in (*O*), along with various mitochondrial markers protein levels in KLEB-infected Fbxo24^+/−^ mice compared to uninfected mice. The densitometric analysis involved calculating the fold change (FC) after normalizing the immunoblot signals to the loading control. Data are mean ± SEM (One-way ANOVA), ∗∗∗∗*p* < 0.001 ∗∗*p* < 0.01 and ∗*p* < 0.05. CFU, colony forming units; FOX, forkhead box.

To assess the indispensability of FBXO24 in mediating FOXK2 effects on mitochondrial function Seahorse assays were conducted in precision cut lung slices isolated from Wt and Fbxo24^+/−^ mice following *K. pneumoniae* infection (Fig. 7). Notably, *K. pneumoniae* infection led to significant reductions in mitochondrial function, including basal respiration, ATP production, maximal respiration, and spare respiratory capacity in Wt mice compared to their uninfected counterparts (Fig. 7, *A*–*E*). However, *K. pneumoniae* infected Fbxo24^+/−^ mice did not exhibit significant reductions in any of the measured mitochondrial parameters when compared to uninfected Fbxo24^+/−^ mice (Fig. 7, *F*–*J*). Further, *K. pneumoniae* infection did not have significant effects on other mitochondrial parameters, such as nonmitochondrial oxygen consumption, proton leak, and coupling efficiency, in either Wt mice (Fig. S9, *A*–*C*) or Fbxo24^+/−^ mice (Fig. S9, *D*–*F*). These results suggest that FBXO24 plays a crucial role in impairing mitochondrial function during infection.

Additional validation studies using genomic PCR using specific primers detected the missing allele within Fbxo24^+/−^ mice (Fig. 7*K*). Immunoblotting was performed in the lungs of Wt mice (Fig. 7, *L* and *M*) and of Fbxo24^+/−^ mice (Fig. 7, *N* and *O*) with and without *K. pneumoniae* infection. The immunoblots were probed for FOXK2 (Fig. 7, *L* and *N*), along with other relevant markers, including OPA1, MFF, PHB1, (Fig. 7, *L* and *N*), Tom20, VDAC, Tom70, Tim23, cytochrome C, and PGC1α (Fig. S9*G*). The results showed that *K. pneumoniae* infection at higher colony forming units (CFU) triggered depletion of FOXK2 protein in lungs of Wt mice coupled with decreased mitochondrial markers (Figs. 7*L*, and S9*G*). In contrast, immunoreactive levels of FOXK2, OPA1, MFF, and PHB1 generally were preserved in the majority of Fbxo24^+/−^ mice regardless of infection (Fig. 7, *N* and *O*). Additionally, we measured extracellular H_2_O_2_ levels in the bronchoalveolar lavage from these mice (Fig. S10). The data revealed a significant increase in extracellular H_2_O_2_ levels in response to *K. pneumoniae* infection in Wt mice but not in FBXO24^+/−^ mice. This finding suggests mitochondrial dysfunction and oxidative stress, as mitochondria are a major source of reactive oxygen species production, including H_2_O_2_, especially during cellular stress such as infection. In summary, the incomplete targeted disruption of FBXO24 in mice is sufficient to partially restore mitochondrial function and key associated bioenergetic proteins during bacterial infection.

## Discussion

This study is the first demonstration of molecular control of FOXK2 abundance *via* ubiquitin-mediated degradation catalyzed by SCF^FBXO24^ that appears crucial in modulating cellular energetics. The new findings here show that (i) FOXK2 is targeted by some virulent pathogens including *P. aeruginosa* and *K. pneumoniae* for degradation through ubiquitin proteasomal processing, (ii) the SCF^FBXO24^ apparatus recognizes FOXK2 as a substrate for polyubiquitylation primarily through multiple ubiquitin linkages, (iii) FOXK2 *via* residues 428 to 478 region are required for molecular interaction with FBXO24 thereby impacting its stability, and (iv) FOXK2 cellular depletion is linked to alterations in global gene expression, but importantly impairs mitochondrial function. We show specific carboxy-terminal molecular signatures within FOXK2 that appear to be important in preserving mitochondrial function. Last, in an experimental pneumonia model *ex vivo* studies suggest that partial disruption of the Fbxo24 gene in mice is sufficient to restore pulmonary mitochondrial function. The data suggest that some bacterial pathogens exploit the ubiquitin machinery to impair energy metabolism by reducing FOXK2 cellular concentrations thereby limiting its transcriptional activity.

FOXK2 exhibits remarkable capacity to shape the gene expression and epigenetic landscape through its role in modulating a diverse array of transcriptional networks involving chromatin engagement, regulation of immune-related genes, and participation in cellular stress responses. These multifaceted biological roles logically position the transcription factor as a target for virulent pathogens that may undermine host cell function. Consequently, microbial targeting of FOXK2 for degradation limits its transcriptional behavior on key downstream genes but also might control host cell epigenetics. Here, we identified that two gram-negative bacterial pathogens, *P. aeruginosa* and *K. pneumoniae,* each were sufficient to reduce cellular concentrations of FOXK2, but by different mechanisms. While *P. aeruginosa* triggered a rapid reduction in FOXK2 protein cellular lifespan through the proteasome, *K*. *pneumoniae* appears to either reduce FOXK2 mRNA synthesis or destabilize its transcript. Our findings that bacterial pathogens stimulate the turnover of FOXK2 to impact expression of downstream genes resembles findings observed in plants where the MYB30-Interacting E3 ligase targets the transcription factor MYB30 after Pseudomonas infection to affect programmed cell death (62).

Few substrates to date have been identified for SCF^FBXO24^ including nucleoside diphosphate kinase A (63), protein arginine methyltransferase 6 (64), and lysine-specific demethylase 1 (LSD1) (65). The observation that LSD1, a chromatin modulator that promotes tumorigenesis is also targeted by FBXO24 adds a layer of complexity to our findings. For example, we cannot exclude the possibility that effects of the F-box protein on mitochondrial disruption occur *via* altered chromatin structure or epigenetic modifications driven by LSD1, rather than by FOXK2 depletion. Nevertheless, here we provide compelling evidence that FOXK2 is a *bona fide* client of FBXO24 using an unbiased screen showing accumulation of FOXK2 levels in cells expressing an inactive FBXO24 mutant compared to cells expressing a Wt FBXO24 as baits in proximal ligations assays. FBXO24-mediated FOXK2 polyubiquitylation, and loss-of-function and gain-of function studies demonstrated an inverse correlation between FOXK2 and FBXO24 protein levels. We also mapped both putative ubiquitylation acceptor sites (K^164R^, K^300R^, K^328R^, and K^633R^) and uncovered a binding domain (aa 428–478) that is enriched with a mix of polar and hydrophobic residues within FOXK2 targeted by FBXO24. Interactions between F-box proteins and their substrates are typically mediated by specific degrons present on the substrate itself, with hypoxia inducible factor 1α and Von Hippel-Lindau protein showing overlap between the docking site and ubiquitylation sites (66), while others exhibit distinct sites, such as the Notch receptor intracellular domain and FBXW7 (45, 67). Interestingly, while the ubiquitylation sites varied in location within the primary sequence of FOXK2, the polyubiquitylated K^328^ site within the FHD domain raises the possibility that the SCF^FBXO24^ complex directly modifies a residue that limits its ability to engage *cis*-acting elements within DNA. Of note, K^633R^ is also subject to SUMOylation (68), highlighting the intricate interplay between these posttranslational modifications in regulating FOXK2 stability and activity. Regarding binding, the putative phospho-enriched domain within FOXK2 (aa155–203) might directly impair FBXO24 accessibility to mediate FOXK2 ubiquitylation, emphasizing the significance of this region in the degradation process. Further interrogation of the relevant phosphorylated residues within this stretch that confer FOXK2 stability requires additional investigation. Whether FOXK2 is ubiquitylated in the nucleus or the cytosol also requires additional investigation. Our findings that ectopically expressed FBOX24 triggered FOXK2 depletion in the nuclear fraction is consistent with existence of components of the degradation apparatus residing partly within the nucleus (59, 60, 61).

We also evaluated comprehensively, the physiological relevance of reduced FOXK2 levels in human epithelia using two strategies: proteomic and transcriptomic screening. Proteomic analysis of FOXK2 pull-down revealed its associations with various proteins relevant to mitochondrial activities and cellular defense against viral and bacterial infections. Correspondingly, transcriptomic analysis showed that FOXK2 silencing resulted in the regulation of genes involved in diverse pathways, all aligned with the common theme of creating a favorable environment for the survival of virulent pathogens. These pathways, including epithelial-mesenchymal transition (69), hypoxia (70), and hedgehog (71) have been previously recognized as upregulated by various pathogens to enhance their survival within host cells (70, 71, 72, 73). While FOXK2 interactions with these pathways have been extensively explored in the context of cancer biology (74), our studies in a nontumorigenic cell model highlights the broad relevance of FOXK2 in influencing these fundamental homeostatic pathways in the native state.

Our studies demonstrate a unique cytoprotective role of FOXK2 in its ability to preserve mitochondrial function. Mitochondrial dysfunction during microbial infection impairs host immune responses, potentially accelerating pathogen virulence (75). Cellular depletion of *FOXK2* in BEAS-2B cells resulted in downregulation of genes associated with mitochondrial metabolism and biogenesis. Findings were also supported by our proteomic analysis revealing FOXK2 interactions with various mitochondrial regulatory proteins. Functional and biochemical assays here show significantly reduced levels of mitochondrial markers and bioenergetic parameters after silencing *FOXK2*. By expressing domain specific *FOXK2* deletional mutants in epithelia, the stretch of residues residing within the carboxy terminal region downstream of FHD domain were indispensable in maintaining mitochondrial activity. Deletion of the FHD domain to variable degrees also impacted the mitochondrial function. The data might suggest that either the transcriptional or epigenetic behavior of FOXK2 requires the DNA binding, nuclear entry, or carboxyl terminus of the protein to sufficiently trigger expression of mitochondrial transcripts that encode for proteins destined to this organelle. In this respect, the stable truncated mutant lacking the FBXO24-ubiquitylation acceptor site (ΔCt mutant, K^633^) exhibited compromised mitochondrial function. This suggests the potential presence of a binding site within this region for another regulatory protein that may assist in maintaining mitochondrial function through its interaction with FOXK2. Alternatively, an attractive hypothesis might be that the K^633^ site undergoes an alternative modification (acetylation, SUMOylation, and so on.) that optimizes its effect on cellular energy stores. Consistent with our results, two studies, demonstrated in a different model that the loss of both FOXK1 and FOXK2 had deleterious effects on mitochondrial metabolism, particularly in the context of insulin regulation in brown preadipocytes (76, 77).

To assess biological relevance of FBXO24 targeting of FOXK2, we pursued studies using Fbxo24^+/−^ mice in a model of experimental pneumonia using *K. pneumoniae*. In separate studies, we observed that *FBXO24* silencing in BEAS2B cells modulates mitochondrial function (unpublished data). Thus, we tested whether *in vivo* silencing of *Fbxo24* in mice infected with *K. pneumoniae* would attenuate adverse effects of the pathogen on FoxK2 protein mass and cellular energetics. Of note, despite extensive breeding we were unable to generate sufficient numbers of Fbxo24^−/−^ mice for this study suggesting issues with fertility. Hence, we focused on using heterozygous (Fbxo24^+/−^) mice that despite infection with *K. pneumoniae* showed generally preserved lung concentrations of FoxK2 and several mitochondrial markers and related function.

Together, these results underscore the biological significance of FOXK2 and its molecular interplay with FBXO24, emphasizing their crucial roles in host defense, particularly concerning bioenergetics. Targeted depletion of FOXK2 *via* accelerated degradation triggered by microbes might significantly undermine integral cytoprotective pathways that are exploited to enhance microbial virulence. FOXK2 disposal mediated by SCF^FBXO24^ during infections might be an opportunity to devise small molecule FBXO24 inhibitors that preserve FOXK2 levels. In this regard, in preliminary studies using a virtual homology structure-based design we have generated a tool compound that antagonizes FBXO24 activity (unpublished observations) that may be suitable for future *in vivo* testing in animal models of experimental pneumonia.

## Experimental procedures

### Cell culture

Human lung primary cells, HBECs, HPAECs, HLF, and HSAECs (American Type Culture Collection [ATCC]) were cultured according to ATCC recommendations. AT1 cells (LONZA) were kindly provided by Dr Englert (OSU), and AT2 were kindly obtained from Dr Mora and Dr Rojas (OSU). Both AT1 and AT2 cells were maintained in epithelial complete medium (Cell Biologics). Human alveolar macrophages were obtained from lung explants from the Comprehensive Transplant Center (CTC) Human Tissue Biorepository at OSU and maintained in RPMI supplemented with 10% fetal bovine serum (FBS), Hepes, L-glutamine, penicillin/streptomycin. BEAS-2B and HEK-293T cell lines were purchased from ATCC. BEAS-2B cells were maintained in HITES media, which consisted of Dulbecco's modified Eagle's medium (DMEM)/F12 supplemented with 10% FBS, insulin, transferrin, hydrocortisone, β-estradiol, Hepes, L-glutamine and penicillin/streptomycin. HEK-293T cells were cultured in DMEM supplemented with 10% FBS, Hepes, L-glutamine, penicillin/streptomycin. The following antibodies were acquired from Cell Signaling Technologies: FOXK2, FOXK1, FOXM1, FOXO4, FOXP3, TOM20 PGC1α, GAPDH, c-Myc, OPA1, MFF, cytochrome C, VDAC, PHB1, Flag, V5, and Tim23, FBXL2, FBXO45, Tom70, and FBXO3 (Santa Cruz Biotechnology). The membranes were developed using WesternBright peroxidase (Advansta) and subsequently imaged using a ChemiDoc Imager (Bio-Rad). All immunoblotting supplies were from Bio Rad. Densitometric analysis was conducted using ImageJ software (NIH, https://imagej.net).

### Generation of BEAS-2B stably expressing FBXO24-V5

Lentivirus was produced in HEK-293T cells packaged with a tetracycline-inducible pSBtet-RP plasmid expressing luciferase or *FBXO24*-V5 and a third generation triple-plasmid system that was subsequently used for transducing BEAS-2B cells. Successfully transduced cells were selected using puromycin, and the resultant stable cell lines were cultured in HITES media. To maintain the stability and purity of the stable cells, puromycin selection was carried out every 3 to 6 months. All tissue culture supplies were purchased from Gibco.

### Generation of FBXO24 KO BEAS-2B cells

CRISPR/Cas9 technology was used to knock out FBXO24 in BEAS-2B cells. Lentivirus was generated in HEK-293T cells using an all-in-one plasmid (lentiCRISPR v2) harboring Cas9 and *FBXO24*-specific sgRNA or a nonspecific sgRNA and a third generation triple-plasmids system for packaging lentivirus. Forty-eight hours post transduction, BEAS-2B cells were flow sorted for mNeon positive cells.

### Generation of FBXO24 heterozygotes in mice

Fbxo24^+/−^ mice were generated using CRISPR/Cas9 technology at the University of Pittsburgh. Briefly, guide RNAs were constructed and tested in blastocysts. The University of Pittsburgh Transgenic and Targeting core facility injected murine fertilized eggs with CRISPR/Cas9 RNA reagents and implanted injected embryos into pseudo-pregnant females. Appropriate guide RNAs generated double-stranded breaks resulting in a 600 bp deletion producing a nonfunctional allele. The generation of the Fbxo24^+/−^ mouse was confirmed by restriction fragment length polymorphism analysis and DNA sequencing.

### Infectious agents

*K. pneumoniae* (KLEB)*, S. aureus* (SA), and *P. aeruginosa* (*P. aeruginosa strain* PA103) were obtained from ATCC, stored at −80 °C, and seeded onto tryptic soy agar plates (Sigma-Aldrich) 2 days before the infection. Individual colonies of each species from tryptic soy agar plates were grown in tryptic soy broth and the cultures were incubated at 37 °C overnight. The bacterial cultures were then diluted 1:10 to 1:20 and incubated for an additional hour, and the bacterial *A*_600_ was determined using a Nanodrop One (Thermo Fisher Scientific). Liquid cultures were further diluted to the desired CFU/ml in cell culture medium without penicillin/streptomycin for *in vitro* treatment.

### Mouse experimental pneumonia

*Fbxo24*^*+/−*^ (*n* = 5 per group) or Wt (*n* = 4 per group) mice from a C57BL/6J background were given 1 × 10^6^ or 1 × 10^7^ CFU *K. pneumoniae* (KLEB)/mouse, or PBS intranasally (i.n.). Mice were euthanized 48 h post infection, and the lung was harvested and processed for preparation of the precision-cut lung slices (PCLS) and total protein purification followed by immunoblotting. Mice were acclimated at the Ohio State University Animal care facility and maintained according to all federal and institutional animal care guidelines and under the OSU institutional Animal care use Committee approved protocol.

### Preparation of mouse PCLS

PCLS were prepared as previously described (78). Briefly, mice were euthanized by CO_2_ asphyxia. After confirmation of death, the lung and bronchus were exposed. Lungs were loaded with 1.5% of UltraPure Low Melting Point Agarose (Thermo Fisher Scientific, Cat#16520100) in sterile medium (DMEM, Gibco) at 50 °C. The trachea was ligated with a thread to retain the agarose inside the lung. The lung was excised, transferred into a tube with PBS and cooled on ice for 30 min to allow the agarose to hard-set. The lobes were dissected and cut with a vibratome (0.30 mm/s; Leica VT 1200) at a slice thickness of 400 μm. The slices were incubated at 37 °C in a tissue incubator with 5% CO_2_ and then washed in sterile medium (DMEM/F-12, Gibco) three times to remove agarose. Followed by an overnight incubation in DMEM/F-12 medium (Gibco) with 10% FBS (Gibco) and 1% antibiotic-antimycotic mixed solution (Gibco), slices were used for functional studies and immunoblotting.

### Hydrogen peroxide production

H_2_O_2_ was measured fluorometrically using the Amplex Red Hydrogen Peroxide Assay Kit (Thermo Fisher Scientific), according to the manufacturer’s instruction. The accumulation of H_2_O_2_ was monitored in the extracellular bronchoalveolar lavage. Fluorescence intensity was measured using a multiplate reader (SpectraMax Gemini) at an excitation wavelength of 530 nm and an emission wavelength of 590 nm at room temperature (RT). The concentration of H_2_O_2_ was determined using a resorufin-H_2_O_2_ standard calibration curve.

#### mRNA analysis

To analyze changes in gene expression, cells were lysed and processed using the RNeasy Plus Kit (Qiagen) according to the manufacturer's protocol. Cells were processed using a RNeasy Plus Universal Mini Kit (Qiagen). Reverse transcription reactions produced complementary DNA with the High-Capacity RNA-to-cDNA (Applied Biosystems). Expression levels were quantified by real time-quantitative PCR using the SYBR Green system.

### Mitochondria function

Mitochondrial OCR and related parameters were assessed using a Seahorse XFe96 Bioanalyzer (Agilent). Cells were transiently transfected with *FOXK2*-specific siRNA or scrambled siRNA 2 days prior to the assay. Twenty-four hours post transfection, cells were counted and 4 × 10^4^ cells per well were seeded into XFe96 cell culture microplates. One hour before the assay, cells were washed twice with XF Cell Mito Stress Test Assay Medium and then incubated at 37 °C without CO_2_. A Seahorse XF Cell Mito Stress Kit (Agilent) was used according to manufacturer’s instructions. In the overexpression experiments, BEAS-2B cells were transiently transfected with a pcDNA3.1 plasmid containing *FOXK2* Wt or mutant plasmids using X-tremeGENE 2 days prior to the assay. The assays were conducted following the same protocol as for the *FOXK2* knockdown cells.

### Site-directed mutagenesis

Mutations were generated using Quikchange Site-Directed Mutagenesis protocol (Invitrogen). FOXK2 internal and carboxy terminal deletions were created using PCR and overlapping primers (Table S2). All primers were designed using SnapGene and synthesized by Integrated DNA Technologies. All plasmids were confirmed by sequencing at the genomic core at OSU and protein expression was validated *in vitro.*

### FOXK2 half-life and its degradation pathway

BEAS-2B cells, either untransfected or 24 h post transfection, were treated with the protein synthesis inhibitor CHX, 50 μg/ml) for 0 to 6 h to determine the half-lives of either endogenous or ectopically expressed *FOXK2*. To investigate FOXK2 degradation pathways, cells were treated with the proteosome inhibitor (MG132, 50 nM), lysosome inhibitor (bafilomycin a1, 20 nM) or a CUL-RING E3 ligase inhibitor (MLN 4924, 5 nM) in the presence or absence of CHX or PA103 (10 MOI) for up to 6 h.

### Immunoblotting

The cells were lysed using protein lysis buffer A (PBS, 0.2% SDS, 0.05% 100X-Triton, and Pierce Protease Inhibitor cocktail, (Thermo Fisher Scientific, A32963)) and sonicated for 20 s at 25% on a Vibra-Cell Sonicator (Sonics). Protein quantification was conducted using Lowry’s assay. The samples were then mixed with Laemmli buffer and separated on SDS-PAGE gels. Afterword, the proteins were transferred to nitrocellulose membranes using the *Trans*-Blot Turbo Transfer system. The membranes were blocked with 5% milk for 30 to 60 min at RT and subsequently incubated overnight with the indicated antibodies. The antibodies used for immunoblotting included anti-FBXO24 (Novus International), anti-β-Actin (Sigma-Aldrich), secondary antibodies (mouse, rabbit, and goat) were obtained from Bio-Rad. The membranes were developed using WesternBright peroxidase (Advansta) and subsequently imaged using a ChemiDoc Imager (Bio-Rad). All immunoblotting supplies were from Bio-Rad. Densitometric analysis was conducted using ImageJ software (NIH).

### FOXK2 pull-down and MS (LC-MS/MS) analysis

To identify FOXK2-associated proteins, HEK-293T cells were transfected with pcDNA3.1-*FOXK2*-Flag using X-tremeGene. Forty-eight hours post transfection, cells were lysed, and protein samples were normalized as described in the immunoblotting section. The lysates were then incubated while rotating at RT for 30 min with anti-Flag M2 Magnetic Beads (Sigma-Aldrich, m8823). Subsequently, beads were washed, and then subjected to on-beads digestion overnight prior to cleanup purification for subsequent analysis using LC/MS-MS. Tandem MS (MS/MS) was carried out on a Thermo Fisher Scientific Orbitrap Fusion MS operating in a MS^3^ mode using synchronous precursor selection for MS^2^ fragment ion selection. MS^2^ peptides sequence data were searched using Mascot Daemon by Matrix Science, version 2.7.0 *via* Proteome Discoverer (version 2.4; Thermo Fisher Scientific, https://www.thermofisher.com/us/en/home/industrial/mass-spectrometry/liquid-chromatography-mass-spectrometry-lc-ms/lc-ms-software/multi-omics-data-analysis/proteome-discoverer-software.html) using UniProt Human database (20210604; 20,513 entries). Peptide identifications were accepted if they could be established at greater than 96.0% probability to achieve a false discovery rate (FDR) less than 1.0% by the Scaffold Local FDR algorithm. Reliable protein identifications, containing at least two unique peptides, were accepted if they could be established at greater than 99.0% probability to achieve an FDR less than 1.0%; probabilities were assigned by the Protein Prophet algorithm (79). For the proteomic results, data are available *via* ProteomeXchange (PXD050221).

### Immunoprecipitation

To determine protein ubiquitylation levels, HEK-293T cells were transfected with pcDNA3.1-*FOXK2*-Flag alone or cotransfected with pcDNA3.1-*FBXO24*-V5, and/or pRK5-*Ubiquitin*-HA (or its mutants). Forty-eight hours post transfection, cells were treated with proteasome inhibitor (MG132) for 6 h to block degradation of ubiquitinated proteins. Next, cells were lysed in lysis Buffer A supplemented with deubiquitinating enzymes and protease inhibitors cocktail and normalized as mentioned earlier in the immunoblotting. The lysates were then incubated while rotating at RT for 30 min with anti-Flag M2 Magnetic Beads (Sigma-Aldrich, m8823). The beads were washed, and then the elution was carried out in 2× Laemmli *via* boiling. Subsequently, the eluted IP and input samples were subjected to immunoblotting. To identify FOXK2 ubiquitinated lysine residues, HEK-293T cells were transfected with pcDNA3.1-*FOXK2*-Flag alone or cotransfected with pcDNA3.1-*FBXO24*-V5. Subsequently, Flag IP was performed, and the beads were subjected to on-beads digestion followed by LC/MS-MS analysis similar to the FOXK2 pull-down method described earlier.

### *In vitro* binding assay

To determine the binding domain of FBXO24 within FOXK2, we generated a series of internal or carboxy-terminal truncated FOXK2-Flag variants. HEK-293T cells were cotransfected with pcDNA3.1-*FBXO24*-V5 and pcDNA3.1-*FOXK2*-Flag Wt or truncated deletion-encoding plasmids for 48 h using X-tremeGENE. Transfected cells were lysed in protein lysis Buffer A by rotating at RT for 30 min, followed by centrifugation at 20,000*g* for 15 min. The denaturing conditions during this type of IP facilitate dissociation of noncovalently bound proteins, ensuring that the detected ubiquitination is covalent. Lysates were then incubated while rotating at RT for 30 min with anti-FlagM2 magnetic beads to pull down FOXK2 and its associated proteins. The beads were washed, and then eluted in 2× Laemmli *via* boiling. The eluted proteins and input samples were separated on SDS-PAGE gels and subsequently subjected to immunoblotting.

### Cell fractionation

BEAS-2B cells were transfected with varying amounts of Flag-tagged FBXO24-expressing plasmid, or V5 tagged *GFP*-expressing plasmid using X-tremeGENE. After 48 h post transfection, subcellular protein fractionation was carried out using the Cell Fractionation Kit (Cell Signaling Technology) following the manufacturer's instructions. Protein concentrations were determined using the bicinchoninic acid assay (Bio-Rad). Lysates were subsequently separated on 4 to 20% gradient SDS-PAGE gels (Bio-Rad) and subjected to immunoblotting. The blots were probed with anti-FOXK2, anti-Flag, anti-V5, anti-GAPDH (cytoplasmic marker), or anti-Lamin A/C (nuclear marker) antibodies.

### Immunofluorescence

BEAS-2B cells were plated at 5 × 10^4^ cells per well on culture slides (Lab-Tek) and allowed to adhere overnight prior to cotransfecting with pcDNA3.1-*FBXO24*-V5 and pcDNA3.1-*FOXK2*-Flag Wt or deletion-encoding plasmids. Transfected or untransfected cells were fixed in 4% paraformaldehyde, permeabilized with 0.5% Triton, and blocked with 5% BSA. Subsequently, cells were stained with primary antibodies including anti-FOXK2 (for endogenous FOXK2), or anti-Flag (ectopically FOXK2), anti V5 (FBXO24) and anti-Tom20 at 4 °C overnight, followed by fluorescent secondary antibody at RT for 4 h. Finally, 4′,6-diamidino-2-phenylindole (DAPI) counterstaining was performed, and images were acquired using an Olympus FV3000 Confocal System microscope.

### Transcriptomic next generation sequencing

To investigate the impact of FOXK2 knockdown in lung epithelia, BEAS-2B cells were transfected with two distinct *FOXK2*-specific siRNA or scrambled siRNA using GenMute. After 72 h post transfection, total RNA was isolated from cells using the RNeasy Plus Kit (Qiagen) according to the manufacturer's instructions. Following RNA extraction with the RNeasy Plus kit, an additional purification step was performed using On-column DNase I (NEB # M0303) treatment for enzymatic removal of residual genomic DNA to ensure RNA quality. RNA quality was evaluated *via* RNA integrity scoring using 2100 Bioanalyzer and/or 2200 TapeStation (Agilent). RNA-seq libraries were generated using kits from New England Biolabs with 100 ng total RNA by targeted depletion of rRNA (NEB E#E6310×). Fragmentation and amplification were carried out using a NEBNext Ultra II Directional (stranded) RNA library Prep kit (NEB#E7760L) and NEBNext (E64490S/L) and Multiplex Oligos for Illumina Unique Dual Index Primer Pairs. The samples were sequenced to a depth of 40 million 2 x 150 bp clusters on the Illumina NovaSeq platform (Illumina, Inc).

### RNA-seq data analysis

We conducted quality control procedures on the raw sequencing data using FastQC v0.12 and MultiQC v1.16 (80). The resulting reads were aligned to the GRCh38 reference genome, and read quantification was carried out in both alignment steps utilizing STAR v2.7.11a (81) with its default settings. Data normalization and differential expression analysis were conducted using the DESeq2 package v3.17 (82). Differentially expressed genes were identified based on adjusted *p*-values (>0.05) and log2 fold change values (>1.5 or < −1.5). To determine the biological significance of these deregulated genes, we performed pathway analysis using the MSigDB Hallmark database through the online platform enrichR (83). Additionally, we evaluated 451 mitochondria-related genes obtained from the Human Gene Set database for both groups. The results were visualized using ComplexHeatmap v3.17 (84), highlighting the most significantly deregulated genes between the groups. Results for RNAseq data have been deposited at GEO (ID: GSE260512).

### Statistical analysis

All data are presented as mean ± SEM and analyzed by one- or two-way ANOVA. Recommended tests for variance of standard deviations were used and associated corrections were used as needed. N indicates the number of independent experiments. The densitometric analysis involved calculating the fold change after normalizing the immunoblot signals to the loading control. Statistical analysis was performed in GraphPad Prism (GraphPad, https://www.graphpad.com/scientific-software/prism/www.graphpad.com/scientific-software/prism/) unless otherwise noted.

## Data availability

Authors will provide all raw data associated with this manuscript upon request. Data are available in Excel, and all full unaltered immunoblots available upon request.

## Supporting information

This article contains supporting information.

## Conflict of interest

The authors declare that they have no conflicts of interest with the contents of this article.
