# Supplementary material for: FOXK2 targeting by the SCF-E3 ligase subunit FBXO24 for ubiquitin mediated degradation modulates mitochondrial respiration
Source: J Biol Chem. 2024 May 10;300(6):107359. doi: 10.1016/j.jbc.2024.107359 (PMC11209018; doi:10.1016/j.jbc.2024.107359)
Supplement: Supporting Table S1 [file mmc1.docx]

| **Protein name** | **Gene name** | **Immune system process (UniProt)** |
| --- | --- | --- |
| Zinc finger CCCH-type antiviral protein 1 | ZC3HAV1 | Is a host antiviral factor that can repress translation and promote degradation of specific viral mrnas |
| 2',5'-phosphodiesterase 12 | PDE12 | Cellular response to interferon-gamma, defense response to virus |
| 40S ribosomal protein S19 | RPS19 | Antimicrobial humoral immune response mediated by antimicrobial peptide, defense response to Gram-negative bacterium, killing of cells of other organism |
| 40S ribosomal protein S30 | FAU | Antibacterial humoral response, antimicrobial humoral immune response mediated by antimicrobial peptide, defense response to Gram-positive bacterium |
| 60S ribosomal protein L30 | RPL30 | Antimicrobial humoral immune response mediated by antimicrobial peptide, defense response to Gram-negative bacterium, killing of cells of other organism |
| Ankyrin repeat domain-containing protein 17 | ANKRD17 | Defense response to bacterium |
| ATP-binding cassette sub-family F member 3 | ABCF3 | Defense response to virus |
| ATP-dependent DNA/RNA helicase DHX36 | DHX36 | Defense response to virus, innate immune response |
| ATP-dependent RNA helicase A | DHX9 | Innate immune response |
| ATP-dependent RNA helicase DDX1 | DDX1 | Defense response to virus, innate immune response |
| Autophagy-related protein 16-1 | ATG16L1 | Defense response to virus |
| Chromodomain-helicase-DNA-binding protein 7 | CHD7 | Response to bacterium |
| Coactosin-like protein | COTL1 | Defense response to fungus |
| DNA dC->dU-editing enzyme APOBEC-3B | APOBEC3B | Defense response to virus, innate immune response |
| DNA mismatch repair protein Mlh1 | MLH1 | Oogenesis, response to bacterium, spermatogenesis |
| DNA-directed RNA polymerase III subunit RPC1 | POLR3A | Defense response to virus, innate immune response |
| DNA-directed RNA polymerase III subunit RPC2 | POLR3B | Defense response to virus, innate immune response |
| DNA-directed RNA polymerase III subunit RPC8 | POLR3H | Defense response to virus, innate immune response |
| Double-stranded RNA-specific adenosine deaminase | ADAR | Defense response to virus |
| E3 ISG15--protein ligase HERC5 | HERC5 | Defense response to virus, innate immune response |
| Endoplasmic reticulum aminopeptidase 1 | ERAP1 | Response to bacterium |
| Glyceraldehyde-3-phosphate dehydrogenase | GAPDH | Antimicrobial humoral immune response mediated by antimicrobial peptide, cellular response to interferon-gamma |
| Glycogen phosphorylase, liver form | PYGL | Response to bacterium |
| Heat shock protein HSP 90-alpha | HSP90AA1 | Activation of innate immune response |
| Helicase MOV-10 | MOV10 | Defense response to virus |
| High mobility group protein B2 | HMGB2 | Cellular response to lipopolysaccharide, defense response to Gram-negative bacterium |
| Histone H2B type 1-J | H2BC11 | Antibacterial humoral response, antimicrobial humoral immune response mediated by antimicrobial peptide |
| Histone H2B type 1-K | H2BC12 | Antibacterial humoral response, antimicrobial humoral immune response mediated by antimicrobial peptide |
| Interferon regulatory factor 3 | IRF3 | MDA-5 signaling pathway, TRIF-dependent toll-like receptor signaling pathway, defense response to virus, type I interferon signaling pathway |
| Interferon-induced, double-stranded RNA-activated protein kinase | EIF2AK2 | Defense response to virus, innate immune response |
| Interleukin enhancer-binding factor 3 | ILF3 | Defense response to virus |
| Mitochondrial import receptor subunit TOM70 | TOMM70 | Activation of innate immune response |
| Nucleolar RNA helicase 2 | DDX21 | Defense response to virus, innate immune response |
| Nucleoporin SEH1 OS=Homo sapiens | SEH1L | Defense response to Gram-positive bacterium |
| Paired amphipathic helix protein Sin3a | SIN3A | Activation of innate immune response |
| Peptidyl-prolyl cis-trans isomerase FKBP5 | FKBP5 | Response to bacterium |
| Poly(rC)-binding protein 2 | PCBP2 | Defense response to virus, innate immune response |
| Pre-mRNA-splicing factor ATP-dependent RNA helicase DHX15 | DHX15 | Defense response to bacterium, defense response to virus |
| Probable ATP-dependent RNA helicase DDX17 | DDX17 | Defense response to virus |
| Probable ATP-dependent RNA helicase DDX56 | DDX56 | Defense response to virus |
| Protein LSM14 homolog A | LSM14A | RIG-I signaling pathway, defense response to virus |
| Proto-oncogene tyrosine-protein kinase Src | SRC | Cellular response to lipopolysaccharide, entry of bacterium into host cell, oogenesis, response to virus, spermatogenesis |
| Ras GTPase-activating protein-binding protein 1 | G3BP1 | Defense response to virus, innate immune response |
| Ras-related protein Rab-14 | RAB14 | Defense response to bacterium |
| Ras-related protein Rab-1A | RAB1A | Defense response to bacterium |
| Ras-related protein Rab-7a | RAB7A | Response to bacterium, viral release from host cell |
| Recombining binding protein suppressor of hairless | RBPJ | Defense response to bacterium, inflammatory response to antigenic stimulus |
| Serine/threonine-protein kinase TBK1 | TBK1 | Defense response to Gram-positive bacterium, defense response to virus, response to virus |
| SHC-transforming protein 1 | SHC1 | Defense response to bacterium |
| Signal transducer and activator of transcription 1-alpha/beta | STAT1 | Defense response to virus, interferon-gamma-mediated signaling pathway, response to interferon-gamma, type I interferon signaling pathway |
| Transcription factor p65 | RELA | Defense response to virus, innate immune response |
| Triadin | TRDN | Response to bacterium |
