# Supplementary material for: FOXK2 targeting by the SCF-E3 ligase subunit FBXO24 for ubiquitin mediated degradation modulates mitochondrial respiration
Source: J Biol Chem. 2024 May 10;300(6):107359. doi: 10.1016/j.jbc.2024.107359 (PMC11209018; doi:10.1016/j.jbc.2024.107359)
Supplement: Supporting Table S2 [file mmc2.docx]

| **Primer Name** | **For Primer** | **Rev Primer** |
| --- | --- | --- |
| **Internal and C terminal deletions** |  |  |
| **∆FHA** | AGGCGGTTCTCCA GACGGCGTGTTCCAGAGGCG | TATCTTGATGTTTGTGCTCGGGAACCTGAATGT |
| **∆155-203** | CACAAACATCAAGATA ATCAGCGCTGCAAACTCCTGCC | TATCTTGATGTTTGTGCTCGGGAACCTGAATGT |
| **∆FHD** | CATCTGACCTCAATTTA TCCCAGGAAGAACCAGGCAAAGGC | TAAATTGAGGTCAGATGGCATCACTCGGC |
| **∆358-378** | CGTGCCCTGC GTGCTGTCTGCTCACTCTAGTGGC | GCAGGGCACGCCCCTAGG |
| **∆428-478** | GCCCCAGGG GCGGTGTCGGTCACCAGTGTGG | CCCTGGGGCGCTCTGGG |
| **∆Ct** | TCAGTCCCCACT TAGTCTAGAGGGCCCGTTTAAACCCGCT | AGTGGGGACTGATGCCACGTG |
|  |  |  |
| **KR** |  |  |
| K128R (aag->aGg) | CGATGTTTGGGC**AGG**AACGGGGTATTCGTGGACGG | TGCCCAAACATCGCAGGTAGAA |
| K171R (aag->aGg) | TGTCCAGCGAG**AGG**AGAGAGAAGCAGGAGGCGTC | TCTCGCTGGACAGGGCAG |
| K174R (aag->agg) | CGAGAAGAGAGAG**AGG**CAGGAGGCGTCTGAGTCTC | TCTCTCTCTTCTCGCTGGACAGG |
| K300R (aaa->aGa) | TTTATACACACATCACT**AGA**AATTATCCCTACTACAGGACTGCGG | TAGTGATGTGTGTATAAATCCCGTTCAGGGT |
| K310R (aag->aGg) | GACTGCGGAC**AGG**GGCTGGCAGAATTCAATTCGC | TGTCCGCAGTCCTGTAGTAGGGA |
| K338R (aaa->aGa) | GAAGAACCAGGC**AGA**GGCTCGTTCTGGAGGATAGAC | TGCCTGGTTCTTCCTGGGAACG |
| K351R (aaa->aGa) | GCCTCTGAAAGC**AGA**TTAATAGAACAGGCTTTTAGGAAACGACGG | TGCTTTCAGAGGCTGGGTCTATCC |
| K422R (aaa->aGa) | TGCACAGCCC**AGA**CTCGCTGTCATCCAGGAAGC | TGGGCTGTGCAGCGCC |
| K537R (aaa->aGa) | GGGAAGTCAAAGTG**AGA**GTAGAGCCTATTCCCGCCATT | TCACTTTGACTTCCCTGTGGTCG |
| K583R (aaa->aGa) | ACCAGCTACCAATA**AGA**ACTGTAACACAAAACGGCACTCAC | TTATTGGTAGCTGGTGTTGACCTAGAGGT |
| K643R (aag->aGg) | CTGAAGCGGATC**AGG**ACAGAAGACGGCGAGGGC | TGATCCGCTTCAGCTCCGG |
