# Supplementary material for: FOXK2 targeting by the SCF-E3 ligase subunit FBXO24 for ubiquitin mediated degradation modulates mitochondrial respiration
Source: J Biol Chem. 2024 May 10;300(6):107359. doi: 10.1016/j.jbc.2024.107359 (PMC11209018; doi:10.1016/j.jbc.2024.107359)
Supplement: Supporting Figures S1–S10 [file mmc3.pdf]

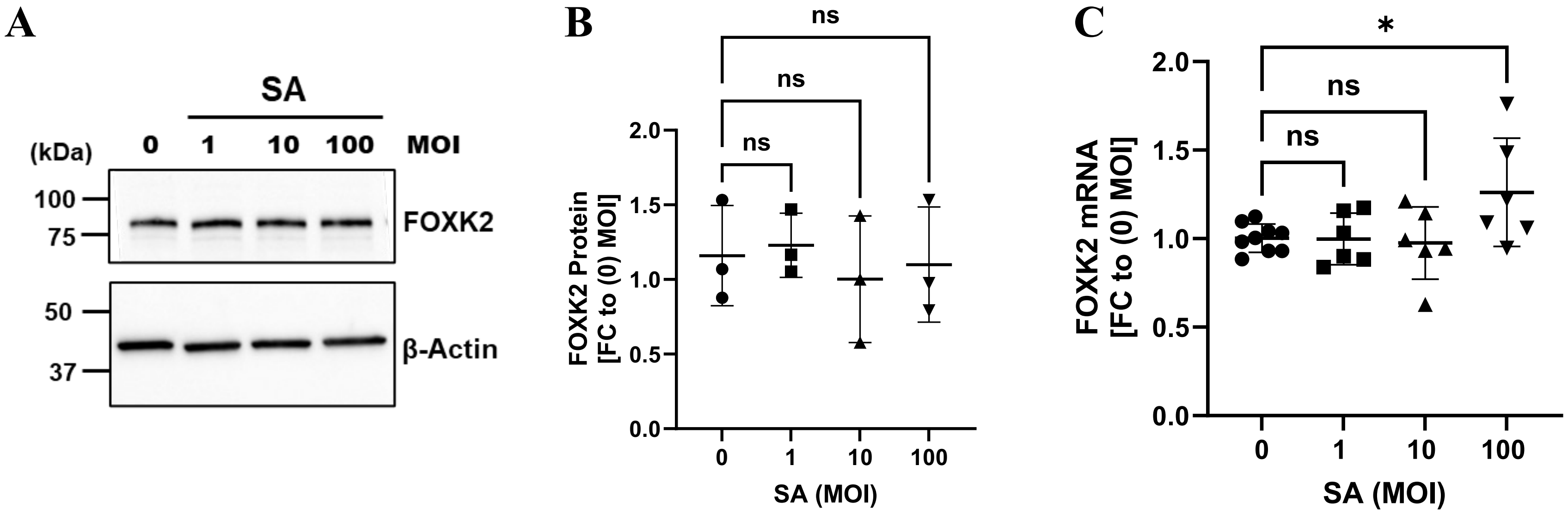

Fig. S1. *S. aureus* (SA) does not alter FOXK2 expression. FOXK2 protein levels in Beas-2B cells following various MOI of SA infection for 6 h. **(A)** Cells were infected with various MOI of *S. aureus* and probed for FOXK2 protein. (**B**) Densitometric quantification of immunoblot data from panel (**A**) for SA infection. Data are expressed as fold-change (FC) relative to MOI=0. **(C)** FOXK2 mRNA levels in Beas-2B cells after *S. aureus* infection for 6 h assayed using qPCR**.**

Supplementary Fig. 1

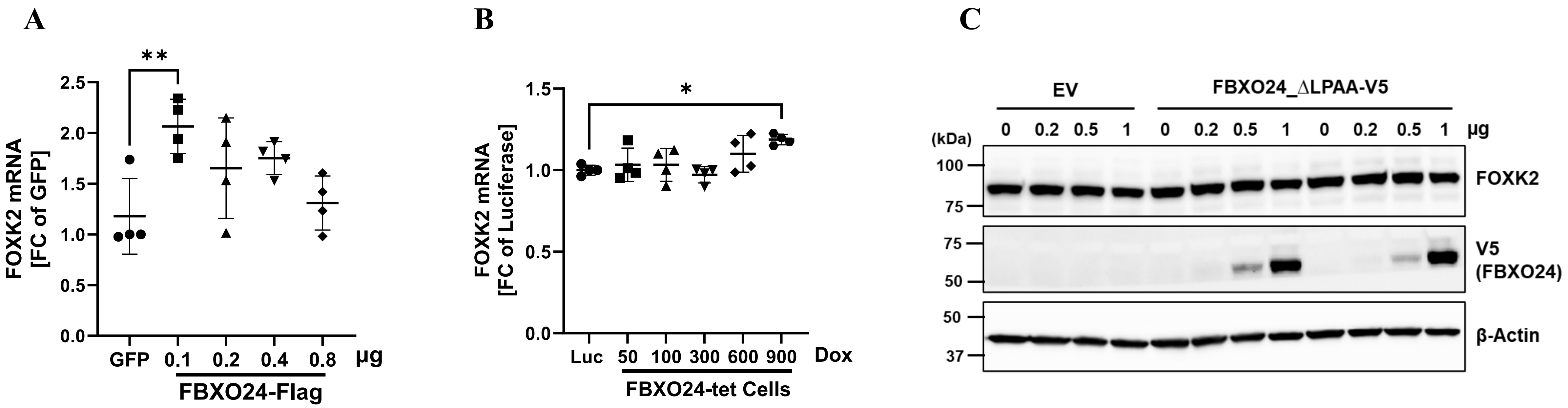

<u>**Fig. S2. FBXO24 does not alter FOXK2 mRNA expression.**</u> *FOXK2* steady-state mRNA levels in Beas-2B cells. (**A**) Cells were transfected with increasing amounts of GFP- or *FBXO24*-Flag plasmid showing no change in steady-state mRNA levels. (**B**) FOXK2 mRNA levels in tetracycline inducible-FBXO24 (FBXO24-tet) Beas-2B cells treated with increasing amounts of doxycycline (Dox) in comparison to luciferase (Luc)-control expressing cells. **(C)** Cells were transfected with increasing amounts of Empty Vector (EV)- or *FBXO24*-ΔLPAA-V5 plasmid showing no change in steady-state FOXK2 protein levels.

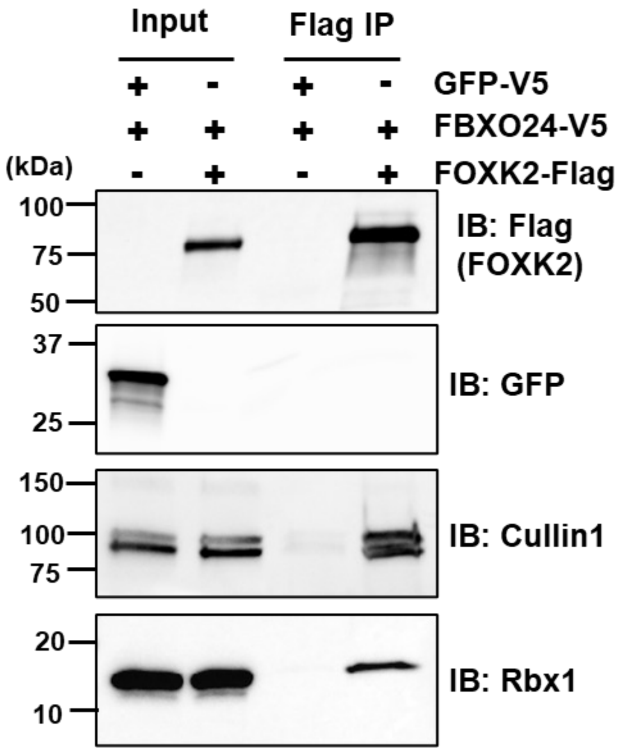

<u>Fig. S3. FOXK2 binds to the SCF ubiquitin machinery.</u> *GFP*-V5 control plasmid or Flag-*FOXK2* plasmid were co-expressed with *FBXO24*-V5 in Beas-2B cells, followed by Co-IP using Flag-beads and immunoblotting. Shown are interactions of SCF components selectively when FOXK2 and FBXO24 are expressed, whereas the SCF components are not detected in association with the expressed control (GFP) suggesting lack of non-specific binding to Flag beads.

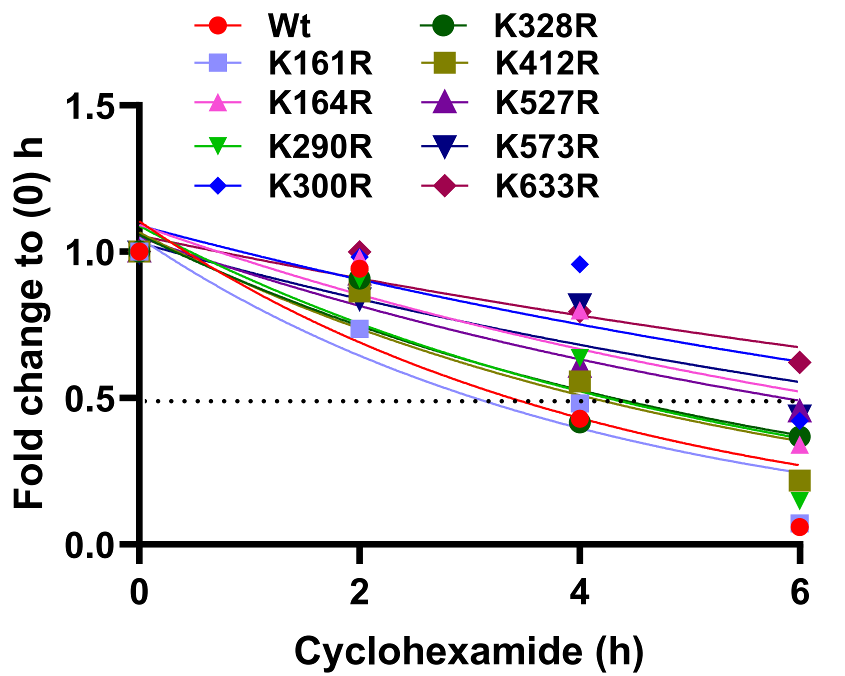

<u>Fig. S4. FOXK2 K-R mutants decay curve.</u> The decay curves showing quantitated levels of various expressed ubiquitin acceptor site mutants expressed in cells that represent data obtained from Fig. 3j.

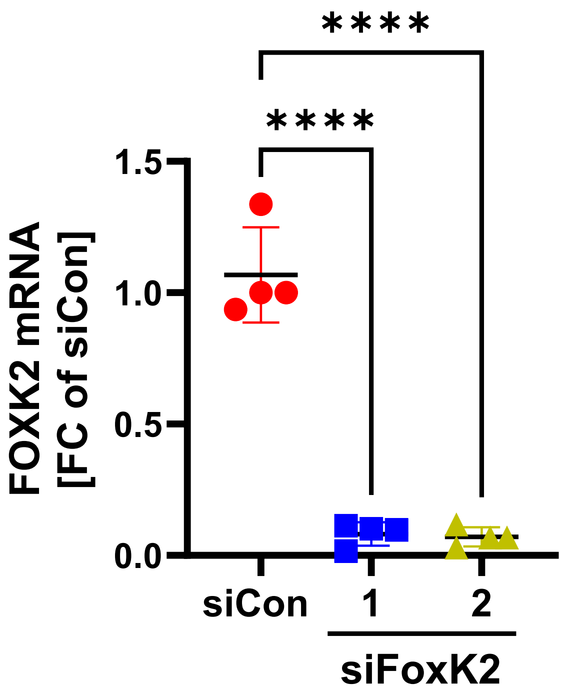

Fig. S5. Silencing of FoxK2. FOXK2 mRNA was assayed confirming successful knockdown of *FOXK2* in Beas-2B cells transfected with predesigned *FOXK2*-specific siRNAs compared to cells transfected with scrambled siRNA.

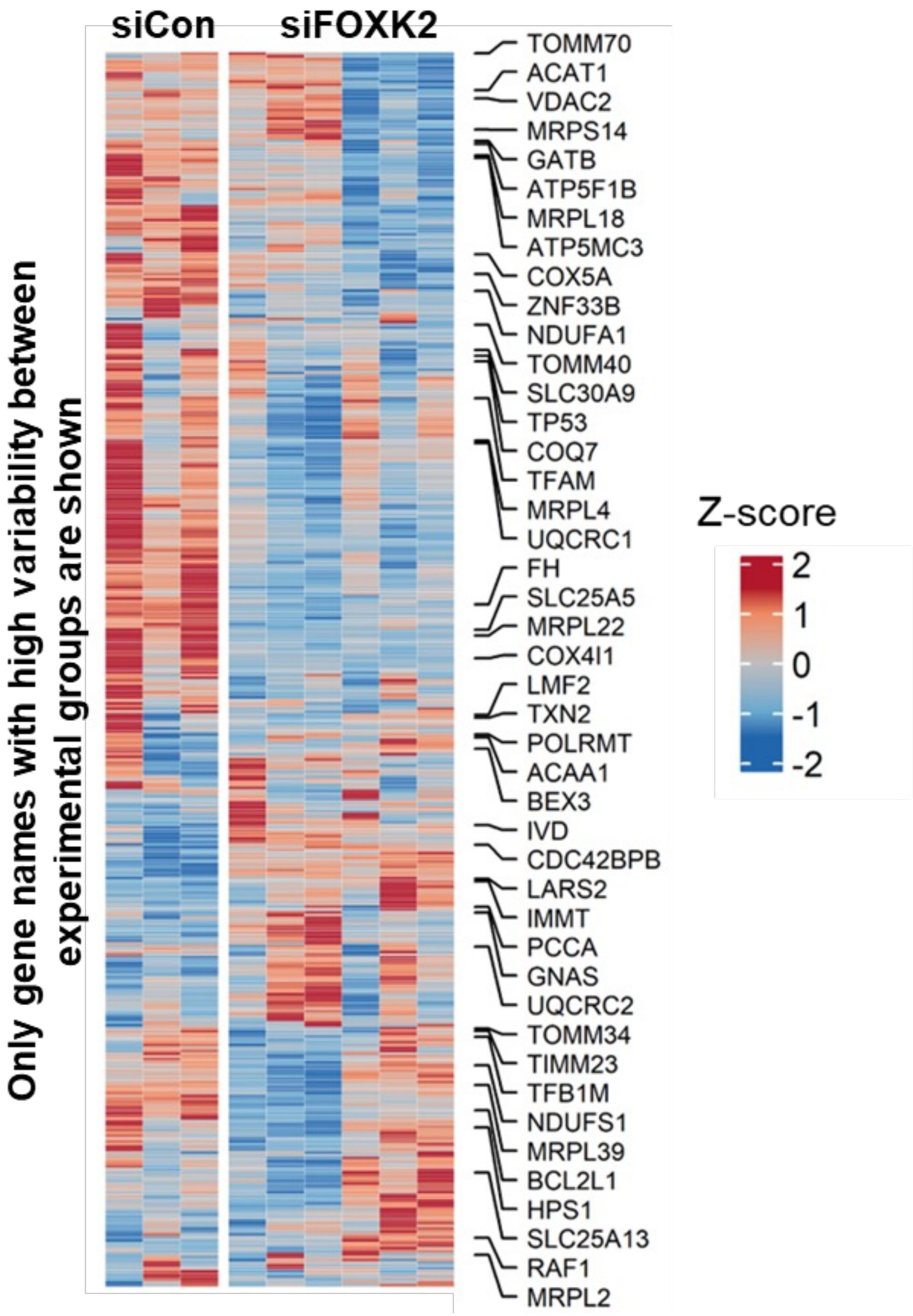

<u>Fig. S6. Heatmap of genes with high variability between control (siCon) versus *FOXK2* knockdown cells.</u> Transcriptomic profiling of *FOXK2*-knockdown cells demonstrating substantial alterations in genes related to protein translation, biogenesis, and the respiratory electron transport.

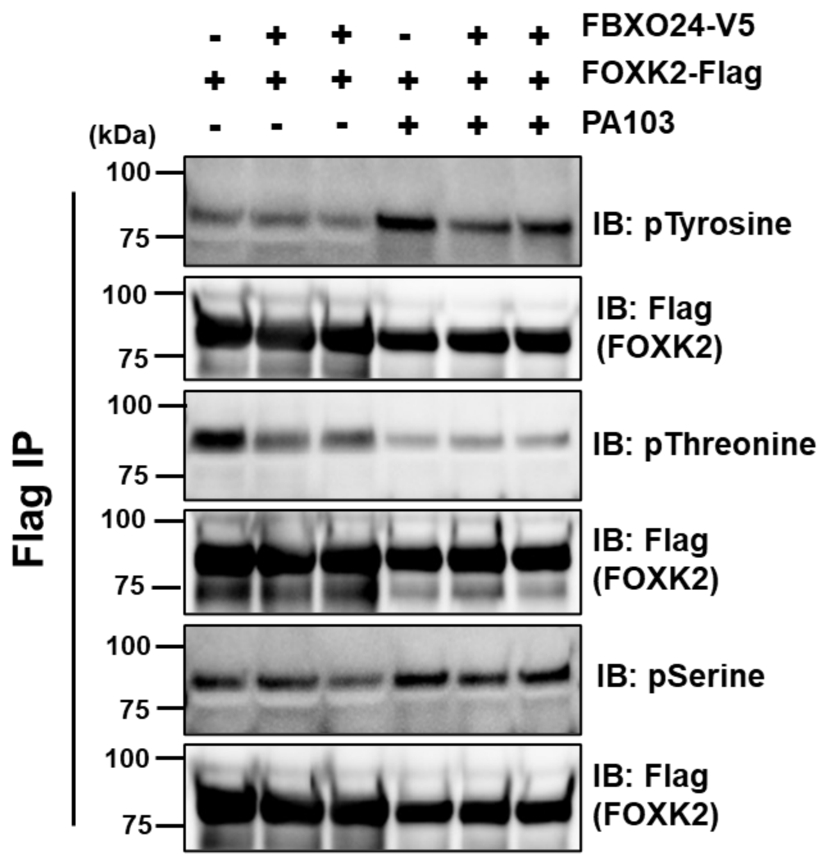

Fig. S7. FOXK2 phosphorylation status during infection. Beas-2B cells were transfected with Flag-*FOXK2* with or without *FBXO24*-V5 followed by bacterial infection. The cells were then processed for Flag immunoprecipitation followed by immunoblotting to assess the phosphorylation levels of FOXK2. The data indicate that *FBXO24* overexpression and PA103 infection differentially modulate the phosphorylation status of FOXK2, affecting different residues in distinct contexts.

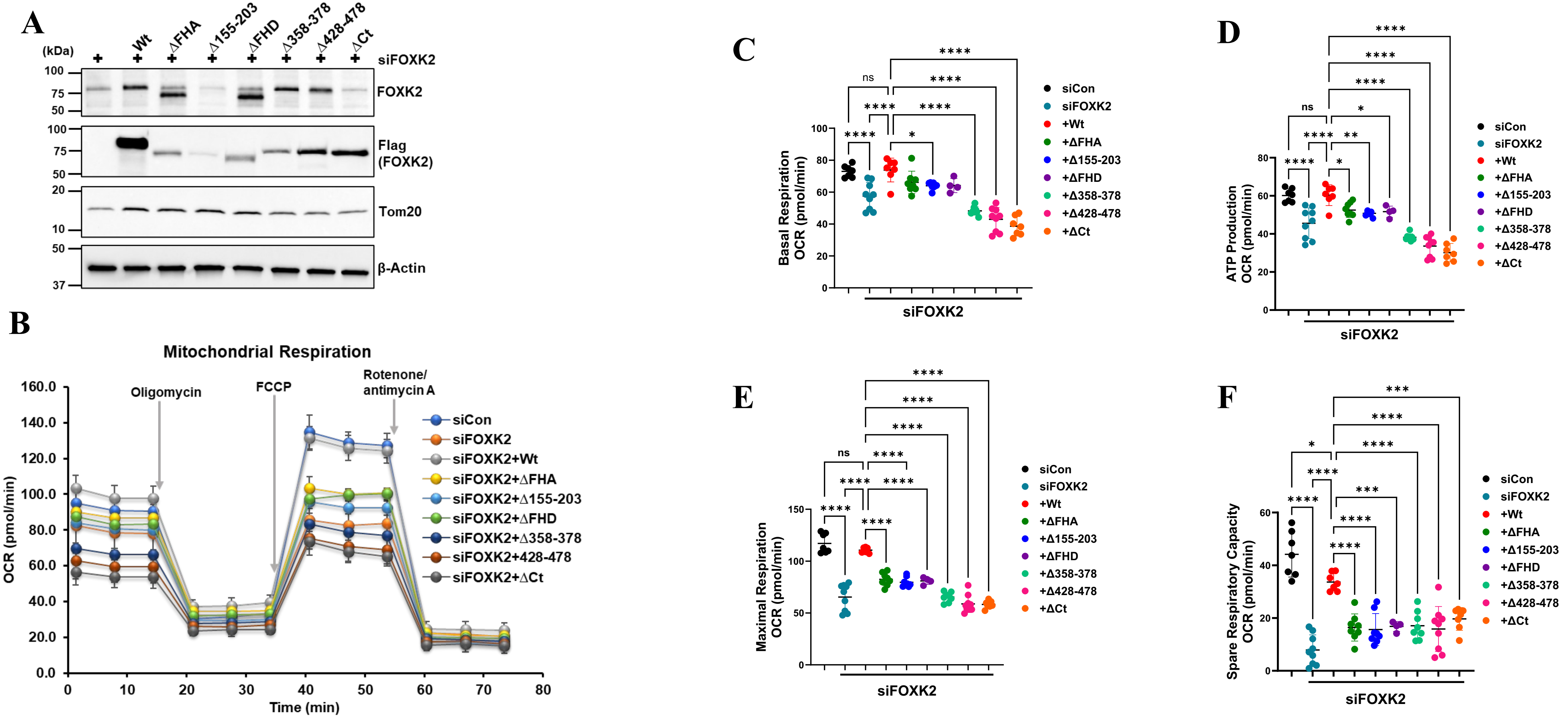

<u>Fig. S8. Depletion of FOXK2 impairs mitochondrial function.</u> Beas-2B cells depleted of endogenous FOXK2 (siFOXK2) were rescued by transfection with internal or carboxyl-terminal truncated mutants. (**A**) Cells were transfected with FOXK2 docking or wild-type (Wt) plasmids and cellular expression assayed by immunoblotting. Mitochondrial oxidative phosphorylation activity (**B**), basal respiration (**C**), ATP production (**D**), maximal respiration (**E**), and spare respiratory capacity (**F**) are shown after cellular expression of plasmids.

Supplementary Fig. 8

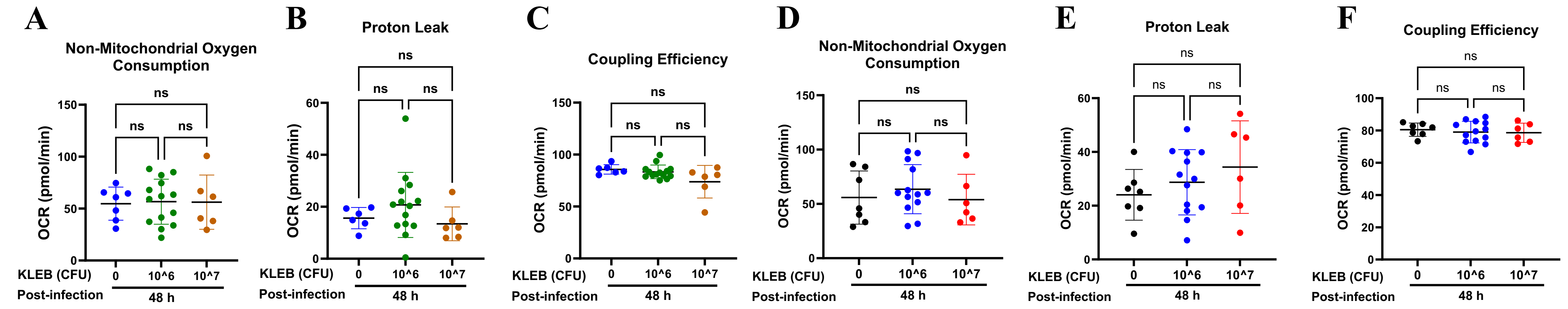

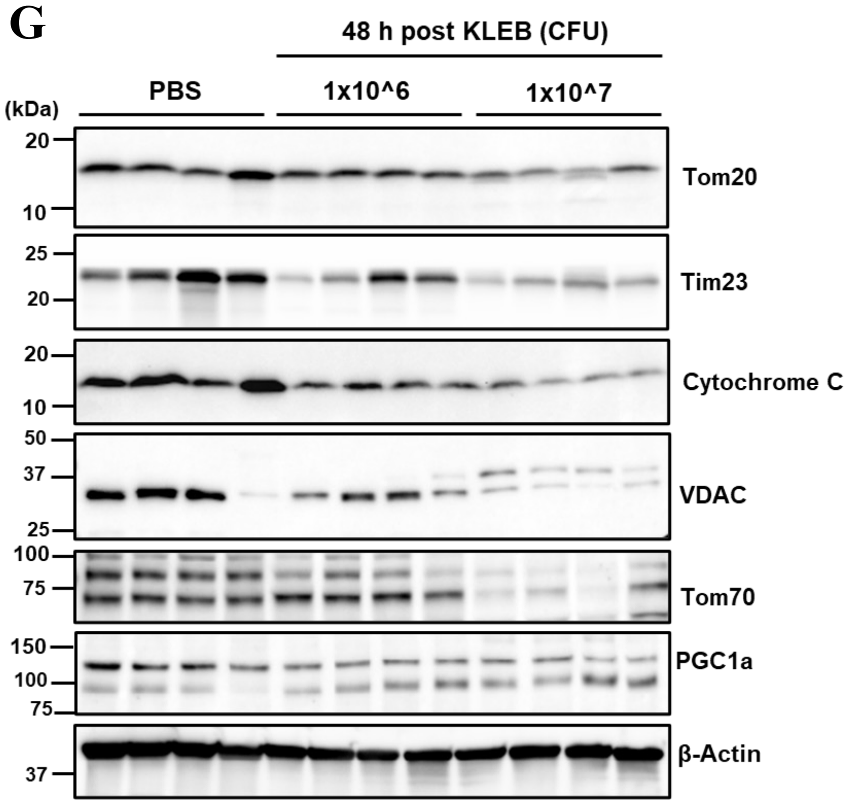

<u>**Fig. S9. Mitochondrial function after *K. pneumoniae* infection.**</u> Both Wt mice (FigS5. 5a-c) or Fbxo24$^{+/-}$ mice (FigS 5d-f) were infected intranasally with or without various CFU/mouse of *K. pneumoniae* (KLEB) and precision lung slices isolated and processed for mitochondrial function (**A-F**) using a Seahorse XFe96 bioanalyzer and immunoblotting for various mitochondrial marker proteins **(G)**.

Supplementary Fig. 9

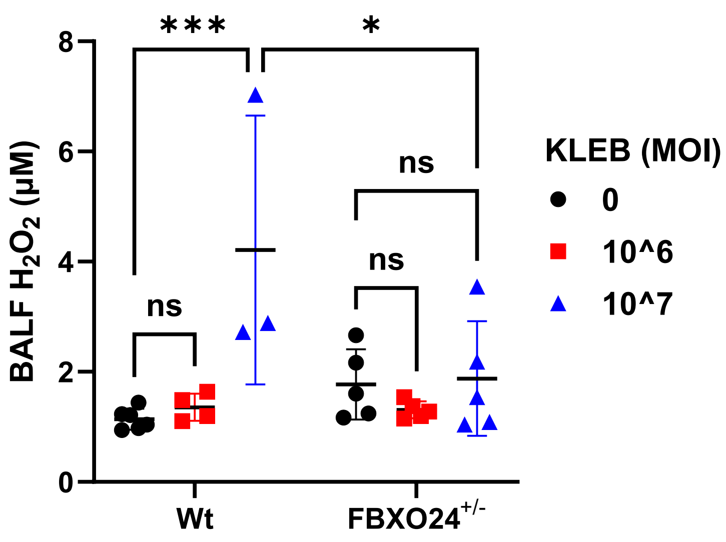

<u>Fig. S10. increased H_2O_2 levels in BALF in Wt mice following KLEB infection.</u> Extracellular H_2O_2 was measured in the BALF from mice presented in Fig. 7. The data revealed a significant increase in extracellular H_2O_2 levels in response to KLEB infection in Wt mice but not in FBXO24$^{+/-}$ mice.

Supplementary Fig. 10
